# Combating *Escherichia coli* O157:H7 with Functionalized Chickpea‐Derived Antimicrobial Peptides

**DOI:** 10.1002/advs.202205301

**Published:** 2022-12-23

**Authors:** Qiao He, Zhehao Yang, Zhipeng Zou, Mengyan Qian, Xiaolei Wang, Xinhui Zhang, Zhongping Yin, Jinhai Wang, Xingqian Ye, Donghong Liu, Mingming Guo

**Affiliations:** ^1^ College of Biosystems Engineering and Food Science National‐Local Joint Engineering Laboratory of Intelligent Food Technology and Equipment Zhejiang Key Laboratory for Agro‐Food Processing Zhejiang University Hangzhou Zhejiang Province 310058 P. R. China; ^2^ Jiangxi Key Laboratory of Natural Products and Functional Foods Jiangxi Agricultural University Nanchang Jiangxi Province 330045 P. R. China; ^3^ Department of Colorectal Surgery The First Affiliated Hospital College of Medicine Zhejiang University Hangzhou Zhejiang Province 310058 P. R. China; ^4^ Fuli Institute of Food Science Zhejiang University Hangzhou Zhejiang Province 310058 P. R. China

**Keywords:** antimicrobial peptides, dual‐targeting mechanism of action, *Escherichia coli* O157:H7, foodborne pathogen intervention, membrane‐mediated antimicrobial mechanism

## Abstract

The rapid dissemination of antibiotic resistance accelerates the desire for new antibacterial agents. Here, a class of antimicrobial peptides (AMPs) is designed by modifying the structural parameters of a natural chickpea‐derived AMP–Leg2, termed “functionalized chickpea‐derived Leg2 antimicrobial peptides” (FCLAPs). Among the FCLAPs, KTA and KTR show superior antibacterial efficacy against the foodborne pathogen *Escherichia coli* (*E. coli*) O157:H7 (with MICs in the range of 2.5–4.7 µmol L^−1^) and demonstrate satisfactory feasibility in alleviating *E. coli* O157:H7‐induced intestinal infection. Additionally, the low cytotoxicity along with insusceptibility to antimicrobial resistance increases the potential of FCLAPs as appealing antimicrobials. Combining the multi‐omics profiling andpeptide‐membrane interaction assays, a unique dual‐targeting mode of action is characterized. To specify the antibacterial mechanism, microscopical observations, membrane‐related physicochemical properties studies, and mass spectrometry assays are further performed. Data indicate that KTA and KTR induce membrane damage by initially targeting the lipopolysaccharide (LPS), thus promoting the peptides to traverse the outer membrane. Subsequently, the peptides intercalate into the peptidoglycan (PGN) layer, blocking its synthesis, and causing a collapse of membrane structure. These findings altogether imply the great potential of KTA and KTR as promising antibacterial candidates in combating the growing threat of *E. coli* O157:H7.

## Graphical abstract

Functionalized chickpea‐derived Leg2 antimicrobial peptides (FCLAPs) show superior antibacterial activity, and they combat *Escherichia coli* O157:H7 through a dual‐targeting mechanism. First, FCLAPs induce membrane damage by initially targeting the lipopolysaccharide, thus promoting the peptides to traverse the outer membrane. Subsequently, the peptides intercalate into the peptidoglycan layer, blocking its synthesis, and causing a collapse of membrane structure.

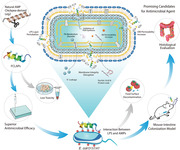

## 1 Introduction

The ever‐growing prevalence of foodborne infections caused by Gram‐negative microbes, especially *E. coli* O157:H7, *Salmonella*, and *Pseudomonas aeruginosa*, has been recognized as a critical healthcare issue that necessitates the development of new antimicrobial agents.^[^ ^1^ ^]^ However, the pipeline for the discovery of novel antimicrobials that target Gram‐negative bacteria remains empty.^[^ ^2^ ^]^ This problem is partly attributed to their intrinsic resistance to most antibiotics that are currently available in clinical applications.^[^ ^3^ ^]^ Another reason is that Gram‐negative bacteria evolved a unique outer membrane (OM) that serves as a highly impermeable barrier to protect themselves from harmful compounds.^[^ ^4^ ^]^ Although recent efforts have been devoted to antimicrobial development, most of the new compounds functioned similarly in their mechanisms to those of traditional antibiotics.^[^ ^5^ ^]^ These worrisome limitations portend the introduction of drugs to clinical applications might be a long and arduous process. Under these circumstances, developing ideal promising antibiotic alternatives that selectively kill Gram‐negative bacteria is imperative to address the urgent medical need.

Antimicrobial peptides (AMPs) have received extensive attention during the past three decades as a new generation of antibiotics.^[^ ^6^ ^]^ Different from most conventional antibiotics that act on specific intracellular targets, AMPs combat bacteria primarily through electrostatic interactions and physically destroying the microbial lipid bilayers.^[^ ^7^ ^]^ The nature of the membrane‐active mechanism renders bacteria with minimal probability to evolve resistance to AMPs, primarily due to the need for a range of genetic mutations to alter the whole components of the bacterial cell membrane.^[^ ^8^ ^]^ At present, more than 3000 AMPs have been documented in the antimicrobial peptide database, however, only seven of them have been approved by the U.S. Food and Drug Administration for clinical application.^[^ ^9^ ^]^ The reduced efficacy in clinically relevant environments, high toxicity toward mammalian cells, and proteolytic instability of natural AMPs were the major challenges that slowed down their clinical implementation.^[^ ^10^ ^]^ and application in the food industry.^[^ ^11^ ^]^ Therefore, new potent AMPs with improved in vivo antimicrobial performance and reduced cytotoxicity are stringently needed to overcome the limitations of natural AMPs.^[^ ^12^ ^]^ In this regard, researchers have been focused on modifying the AMPs isolated from natural sources for stronger properties.^[^ ^13^ ^]^

Great efforts have been made to explore the relationships between the physicochemical parameters of AMPs (i.e., net charge, hydrophobicity, amphiphilicity, and structural propensity) and the antibacterial efficacy which provide references for rational modification and optimization of AMP properties.^[^ ^14^ ^]^ The pioneer studies suggested that fine‐tuning the hydrophobicity and cationic residues of natural AMPs to reach a balanced antimicrobial potential and biocompatibility could be extended to mine new antimicrobial agents.^[^ ^15^ ^]^ Such optimized AMPs would be particularly appealing as they would not only actively kill bacteria but also be potentially useful in healthcare settings. However, the precise mechanisms of action and their antimicrobial targets are barely known or just empirical.^[^ ^9^ ^]^

**Figure 1:**
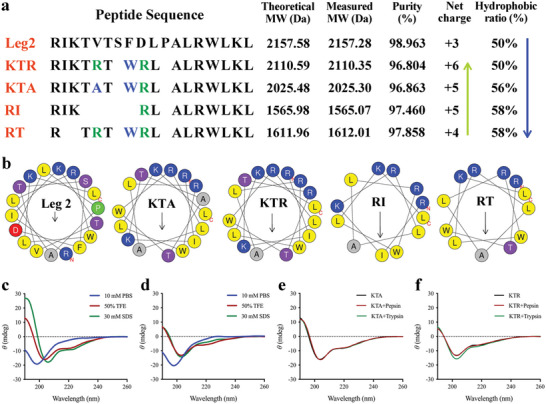
Design, characterization, secondary structure, and protease stability of the Functionalized Chickpea‐derived Leg2 Antimicrobial Peptides (FCLAPs). a) The amino acid sequences, theoretical MWs, measured MWs, purities, net charges, and hydrophobic ratios of the natural peptide (Leg2) and FCLAPs. b) Helical wheel projections of the natural peptide and FCLAPs. c,d) Circular dichroism (CD) spectra of KTA and KTR, respectively. The peptides were dissolved in 10 mmol L^−1^ phosphate‐buffered saline (PBS; pH 7.4), 50% (v/v) trifluoroethanol (TFE), or 30 mmol L^−1^ sodium dodecyl sulfate (SDS). The peptide concentrations were fixed at 0.1 mmol L^−1^. e,f) CD spectra of KTA and KTR treated with the proteases. The peptide concentrations were fixed at 0.1 mmol L^−1^. All of the samples were dissolved in 50% TFE. The peptide/protease molar ratio was 20:1.

**Scheme 1:**
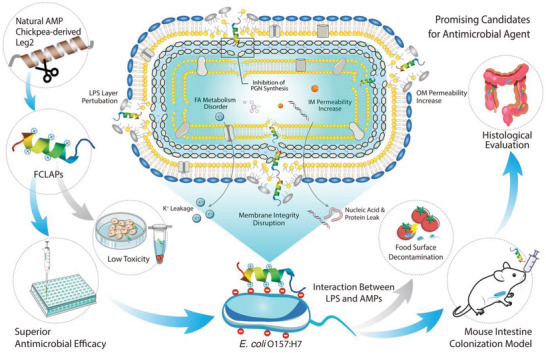
Schematic illustration of FCLAPs combating *E. coli* O157:H7 both in vitro and in vivo. FCLAPs kill bacteria through a dual‐targeting mechanism: 1) interaction with the LPS leaflet of the outer membrane, causing areas of destabilization and facilitating the entry of FCLAPs; 2) FCLAPs further insert into the PGN layer, blocking the PGN synthesis, resulting in a disrupted cell wall architecture. The rupture of cell envelope further induces metabolic disorder of membrane components, leakage of cytoplasmic content, and eventually causes bacterial death.

By manipulating the net charge, hydrophobicity, and sequence length of Leg2 (RIKTVTSFDLPALRWLKL), an AMP derived from a chickpea, we designed the functionalized chickpea‐derived Leg2 antimicrobial peptides (FCLAPs) with varying physiochemical properties (**Figure** **1**). Among the FCLAPs, KTA (RIKTATWRLALRWLKL) and KTR (RIKTRTWRLALRWLKL) showed superior antibacterial potency both in vitro and in vivo. Further, an integrative analysis of the transcriptomics and metabolomics profiling as well as a panel of membrane property analyses were conducted to investigate their inactivation mechanisms of action. Based on the data collected, we put forward a plausible mechanism whereby FCLAPs initially approach LPS leaflet and then destabilize the outer membrane via peptide‐LPS interaction; Upon interaction, the metabolic state of membrane lipids alters and the membrane architecture disrupts, thus facilitating peptides intercalate into the PGN layer and block its synthesis to exert bactericidal activities (**Scheme** **1**). To the best of our knowledge, this is the first proposal of a unique dual‐targeting mechanism underpinning the superior antimicrobial activity of AMPs. We also determine the role of PGN as an antibacterial target, filling up the lack of insight into the AMP‐peptidoglycan interaction. Altogether, our findings suggest that KTA and KTR represent promising sources of antibacterial candidates that could aid efforts to fill the drug discovery pipeline as a defense against Gram‐negative foodborne bacteria.

## 2 Results

### 2.1 Design and Characterization of FCLAPs

In this study, a natural AMP Leg2 (RIKTVTSFDLPALRWLKL) from a chickpea was used as the parental peptide.^[^ ^16^ ^]^ To improve the antibacterial activity of Leg2, we developed rational modification and optimization of its properties. In this regard, the main factors that affect the potent activity of AMP including cation, hydrophobicity, and sequence length were taken into consideration. The amino acid sequences and characterization data are shown in Figure 1 and Figure S1, Supporting Information. Concretely, we first replaced the Asp on site 9 of Leg2 with a positively charged amino acid residue arginine (Arg) in FCLAPs to increase the net charges. Considering that the hydrophobic amino acid residues aid in penetrating the hydrophobic core of bacterial membranes, tryptophan (Trp) was introduced into FCLAPs and replaced phenylalanine (Phe) on position 8. The proline (Pro) and serine (Ser), which were not conducive to forming the *α*‐helical structures, were deleted on the Leg2. Next, in view of the strong hydrophobicity that might increase cytotoxicity, the valine (Val) on site 5 was substituted by alanine (Ala) and Arg to obtain the two peptides KTA and KTR, respectively. On this basis, peptides RI (RIKRLALRWLKL) and RT (RTRTWRLALRWLKL) were generated by shortening the amino acid sequence of KTA and KTR. The synthesized FCLAPs were characterized by triple‐quadrupole time‐of‐flight mass spectrometry (Q‐TOF MS) and reversed‐phase high‐performance liquid chromatography (RP‐HPLC). As shown in Figure 1a and Figure S1, Supporting Information, the measured molecular weights (MWs) of the FCLAPs were close to their theoretical values, and the purities were higher than 95%, indicating that the peptides were synthesized to the desired specifications.

According to the helical wheel projections predicted online, the FCLAPs showed a strong tendency to form highly amphipathic *α*‐helical structures (Figure 1b). In this regard, circular dichroism (CD) spectroscopy was performed to evaluate the global conformations of the FCLAPs in different environments. As shown in Figure 1c,d and Figure S2, Supporting Information, the FCLAPs presented unstructured conformations in phosphate‐buffered saline (PBS). In sodium dodecyl sulfate (SDS) micelle and 50% trifluoroethanol (TFE) solution, which were employed to mimic the anionic and hydrophobic membrane environment, the FCLAPs adopted well‐defined *α*‐helical structures, as demonstrated by the presence of the characteristic double minima at approximately 208 and 222 nm.

### 2.2 In Vitro Antibacterial Activity and Cytotoxicity Assays

To evaluate the antibacterial efficacy of FCLAPs, their minimum inhibition concentrations (MICs) against a wide spectrum of Gram‐negative (*Escherichia coli*, *Salmonella Typhimurium*, *Klebsiella pneumoniae*, *Acinetobacter baumannii*, *Pseudomonas aeruginosa*, *Enterobacter cloacae*) and Gram‐positive (*Enterococcus faecium*, *Staphylococcus aureus*, and Methicillin‐resistant *Staphylococcus aureus*) bacteria were determined. Encouragingly, FCLAPs showed potent broad‐spectrum antibacterial activity, with MICs in the range of 2.5–31.9 µmol L^−1^ against most of the strains tested (**Table** **1**). The antibacterial activities were strikingly improved as compared with the parental peptide Leg2 (MICs ≥ 370.8 µmol L^−1^). According to the literature, the low MICs of FCLAPs might be attributed to their increments in cationic and hydrophobicity.^[^ ^17^ ^]^ Among them, the MICs of KTR against most of the bacteria tested were found to be within the same order of magnitude (2.4 – 9.5 µmol L^−1^), indicating that the antibacterial efficacy of KTR might not be species‐specific. This was surprising, as *Klebsiella pneumoniae* and *Pseudomonas aeruginosa* were theoretically more resistant due to the production of thick extracellular capsules which inhibited the penetration of antimicrobial agents.^[^ ^18^ ^]^ It was also noteworthy that KTA and KTR showed preferential bacteriostatic activity towards *E. coli* O157:H7, yielding MIC values ranging from 2.5 to 9.6 µmol L^−1^. Especially for KTA, its performance against *E. coli* O157:H7 was even superior to that of colistin, which is acknowledged as the last line of defense against Gram‐negative pathogens. But the RI and RT fell short of KTA and KTR in antibacterial potent, suggesting that a certain balance between cationicity, hydrophobicity, and length is required to reach the optimal activity.

**Table 1:** Antibacterial activities of the natural and functionalized chickpea‐derived Leg2 antimicrobial peptides (FCLAPs)

Organisms	MIC [µmol L^−1^]						
	KTA	KTR	RI	RT	Leg2^a^	Col^b^	Tob^c^
*Escherichia coli* O157:H7 ATCC 35150	2.5	4.7	9.6	6.2	370.8	3.2	8.6
*Escherichia coli* ATCC 8739	4.9	4.7	12.8	15.5	370.8	3.2	4.3
*Escherichia coli* MDR^d^	4.9	4.7	16.0	6.2	> 463.5	102.1	547.6
*Salmonella Typhimurium* ATCC 14028	9.9	4.7	31.9	15.5	> 463.5	6.4	17.1
*Klebsiella pneumoniae* ATCC 13883	12.3	2.4	63.9	12.4	> 463.5	12.8	2.1
*Acinetobacter baumannii* ATCC 17978	4.9	2.4	12.8	6.2	> 463.5	1.6	2.1
*Pseudomonas aeruginosa* ATCC 10145	49.4	9.5	31.9	31.0	> 463.5	6.4	4.3
*Enterobacter cloacae* ATCC 13047	98.7	118.5	191.6	186.1	> 463.5	204.2	2.1
*Enterococcus faecium* ATCC 19434	49.4	47.7	95.8	62.0	> 463.5	204.2	273.8
*Staphylococcus aureus* ATCC 25923	9.9	9.5	127.4	31.0	> 463.5	12.8	17.1
*Staphylococcus aureus* MRSA ATCC 43300	7.4	7.1	63.0	15.5	> 463.5	51.1	547.6

^a)^ Leg2, the natural parental antimicrobial peptide

^b)^ Col, colistin

^c)^ Tob, tobramycin. KTA, KTR, RI, and RT stands for RIKTATWRLALRWLKL, RIKTRTWRLALRWLKL, RIKRLALRWLKL, and RTRTWRLALRWLKL, respectively

^d)^ multi‐drug resistant clinical isolate.

We next examined the antibacterial potential of FCLAPs by measuring the MICs of the multi‐drug resistant bacteria (i.e., *E. coli* MDR and MRSA). The results showed that the FCLAPs demonstrated essentially equipotent activity against drug‐sensitive strains as well as the MDR ones. Especially for KTA and KTR, their MICs against *E. coli* MDR and *Staphylococcus aureus* MRSA were in the range of 4.7 to 7.4 µmol L^−1^. As a comparison, the antimicrobial activities of tobramycin and colistin, clinically‐used antibiotics known to be effective against Gram‐negative pathogens, were also evaluated. Both tobramycin and colistin were found to be poorly active against *E. coli* MDR, with MICs at least 128‐ or 32‐fold higher than those of wild‐type *E. coli* strains, respectively.

**Figure 2:**
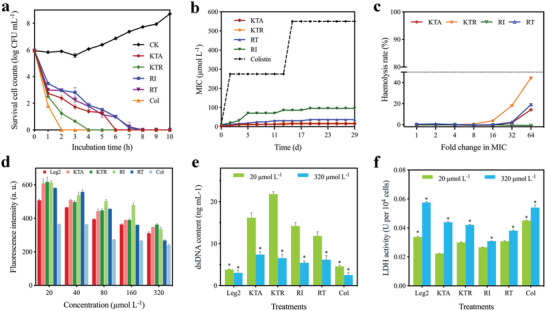
In vitro antibacterial activity and biosafety of the FCLAPs. a) Time‐dependent killing of *E. coli* O157:H7 by FCLAPs. A stationary phase culture of *E. coli* O157:H7 was challenged with 4 × MIC AMPs. Colistin (4 × MIC) was used as a positive control, and sterile PBS was used as a negative control. *n* = 3 biologically independent samples. Data were mean ± SD. b) Development of bacterial resistance towards the FCLAPs and colistin was evaluated using MIC test over time. The curves show representative examples of *n* = 3 biologically independent experiments c) Haemolytic activity of the FCLAPs to the red blood cells of SD rat. 1% Triton X‐100 was used as a positive control, and PBS was used as a negative control. d) Cytotoxicity of Leg2 and FCLAPs was evaluated using Alamar Blue assay on LO2 cell line. e) Cell proliferation was assessed by PicoGreen assay to quantitatively measure the double‐stranded DNA content of LO2 cells after FCLAP treatments. The content of double‐stranded DNA of untreated cells was 22.65 ± 0.88 ng mL^−1^. f) Cytotoxicity of FCLAPs was evaluated by the activity of lactate dehydrogenase (LDH) released from the cytoplasm of LO2 cells. The activity of LDH released from the untreated cells was 0.02778 ± 0.0005. Colistin was used as a positive control in (d–f). Experiments in (a–f) were performed as three biologically independent experiments, data presented as mean ± SD, *n* = 3. Data between treated and untreated cells in (e‐f) were analyzed using an unpaired Students’ *t*‐test. * indicates *P* < 0.0001.

Furthermore, the antibacterial activities of FCLAPs were examined by the minimal bactericidal concentration (MBC) assays.^[^ ^19^ ^]^ As illustrated in Table S1, Supporting Information, the values of MBC were the same or less than fourfold of their corresponding MICs, demonstrating that such FCLAPs have favorable bactericidal activity. Of note, KTR exhibited better bactericidal efficacy against *E. coli* O157:H7 than KTA, as its relatively lower MBC value (7.1 µmol L^−1^ for KTR and 9.9 µmol L^−1^ for KTA). This result, in contrast to the MIC, suggested that KTA was more effective in inhibiting the growth of *E. coli* O157:H7, while KTR appeared more potent than KTA in bacterial killing. As expected, KTR and KTA killed almost all *E. coli* O157:H7 cells within 4 and 6 h at their 4 × MIC concentrations, respectively, exhibiting potent and rapid bactericidal efficacy (**Figure** **2**a). Consistent with the results of MIC and MBC, the time‐kill curves added to the evidence that KTA and KTR could emerge as promising bactericidal candidates.

To further evaluate if resistance against FCLAPs could be developed easily, we studied the bacterial behavior in response to repeated long‐term exposure to the peptides. As illustrated in Figure 2b, the MIC values of the KTA and KTR towards *E. coli* cells remained relatively constant throughout the tests (over 30 days). Conversely, rapid acquisition of resistance was elicited in colistin‐treated *E. coli* O157:H7 cells by the second cultivation step, with the MIC rising to 32 times the initial value. That is, KTA and KTR were less prone to evolve de novo resistance under the tested conditions, as compared with the antibiotic colistin.

To assess whether the FCLAPs were resistant to the digestive proteases, we first tested the antibacterial activities of the peptides incubated with various levels of pepsin or trypsin. Melittin, a typical *α*‐helical AMP that is in clinical use as an alternative to antibiotics, was used as a positive control. As illustrated in Table S2, Supporting Information, all the FCLAPs retained their effective antibacterial activities after incubation with pepsin. However, in the case of trypsin, the antibacterial activities of FCLAPs were slightly compromised by the high concentration of trypsin. This phenomenon could be explained by high Lys and Arg contents in the FCLAPs, which were potentially digested by high concentrations of trypsin. In contrast, melittin was completely inactivated in the presence of a low concentration of pepsin or trypsin. The results demonstrated that the FCLAPs were more resistant to digestive proteases than melittin.

Subsequently, a CD measurement was performed to explore the conformation alteration of the FCLAPs after the digestive protease treatments. As shown in Figure 1e,f and Figure S3, Supporting Information, all the FCLAPs treated with pepsin or trypsin exhibited similar structural tendencies to the peptides alone. However, melittin showed enormous secondary structural changes compared to its typical *α*‐helical structure. We also conducted an HPLC analysis to determine the composition and content of the peptides in the presence of proteases. Similar HPLC profiles to the controls (peptides alone) were observed in the protease‐treated ones (Figure S4, Supporting Information). Unsurprising, the degradation of melittin was presented, as indicated by the increase in the number of chromatographic peaks and the decrease in the response value of the target peak. Together, the proteolytic stability studies indicated that the FCLAPs possess great resistance to digestive proteases.

As a test of in vitro cytocompatibility, the toxicity of FCLAPs was evaluated using MTT assays by incubating them with cell lines derived from normal tissues including mouse embryo cells (NIH/3T3 cells) and human normal liver cells (LO2 cells). FCLAPs exhibited similarly low cytotoxicity toward the two types of mammalian cells within the range of concentrations tested (Figure S5, Supporting Information). In the case of LO2 cells, the survival rates were 86.7% and 83.7% in response to KTA and KTR, respectively, even at the highest tested concentration of 640 µg mL^−1^. Colistin, as a positive control, displayed slightly higher cytotoxic activity toward the two cell lines.

Further, the viability of the LO2 cells treated by FCLAPs was evaluated using the Alamar Blue assay. As shown in Figure 2d, the fluorescence intensity tended to slightly decrease with the increase in peptide dosage, which might be caused by the decrease in the cell metabolic activity upon a high concentration of FCLAPs. Then, the PicoGreen assay was performed to quantitatively measure the double‐stranded DNA (dsDNA) content of LO2 cells after treatments as an assessment of cell proliferation.^[^ ^20^ ^]^ It was found that FCLAPs produced a smaller decrease in dsDNA content compared with colistin, indicating that FCLAPs exhibit less effect on cell proliferation (Figure 2e). Another method for determining cytotoxicity is based on assessing the activity of lactate dehydrogenase (LDH). It is a cytoplasmic enzyme that would rapidly leak into the cell culture supernatant when the cell membrane is destroyed.^[^ ^21^ ^]^ The results showed that cells treated with FCLAPs released less LDH to solutions, indicating a lower number of damaged cells (Figure 2f). In the above cytotoxicity assays, it was found that the toxicity of FCLAPs was much lower than the parental peptide Leg2. It was not unexpected since the high cytotoxicity of natural AMPs as a major obstacle that impeded their clinical translational development has been reported in plenty of studies.^[^ ^22^ ^]^ The results demonstrated that the peptide modification strategy effectively decreased the cytotoxicity of mammalian cells.

Subsequently, we evaluated the hemolytic activities of FCLAPs by investigating their effect on the rupture and lysis of red blood cells. The results showed that RI induced negligible hemolysis (0.78%) even at 613.0 µmol L^‐1^ m (960 µg mL^−1^). Even at a very high concentration of 32 × MIC, the extent of hemolysis was well below 20%, indicating the excellent blood compatibility of FCLAPs (Figure 2c; Figure S6, Supporting Information). Illustrated by the above results, FCLAPs are promising candidates to combat Gram‐negative bacteria *E. coli* O157:H7, thus prompting us to test their antibacterial potential for in vivo application.

### 2.3 In Vivo Antimicrobial Efficacy

**Figure 3:**
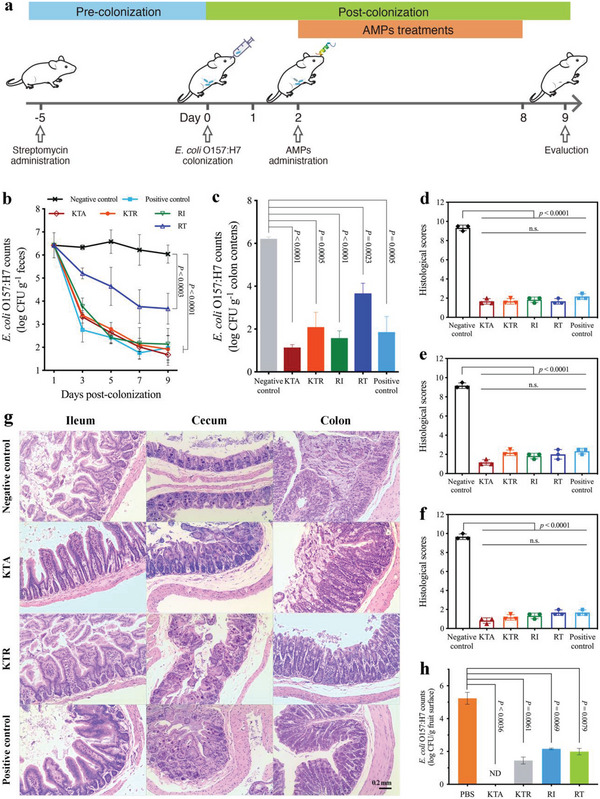
FCLAPs exerted great therapeutic potential in in vivo models. a) Schematic diagram of experiment design (not drawn to scale). b) *E. coli*‐colonized mice model. KTA (20 mg kg^−1^, 9.87 µmol L^−1^), KTR (40 mg kg^−1^, 18.95 µmol L^−1^), RI (60 mg kg^−1^, 38.31 µmol L^−1^), and RT (40 mg kg^−1^, 24.81 µmol L^−1^) rapidly decreased fecal number of *E. coli* O157:H7 (2.0 × 10^8^ CFUs) after *E. coli* colonization. PBS was used as a negative control. Ciprofloxacin (10 mg kg^−1^, 30.18 µmol L^−1^) was used as a positive control. The limit of detection was approximately 1 × 10^1^ CFU g^−1^ feces. c) KTA (20 mg kg^−1^, 9.87 µmol L^−1^), KTR (40 mg kg^−1^, 18.95 µmol L^−1^), RI (60 mg kg^−1^, 38.31 µmol L^−1^), and RT (40 mg kg^−1^, 24.81 µmol L^−1^) rapidly decreased the bacterial loads of *E. coli* O157:H7 in the colon after treatment for 7 days. PBS was used as a negative control. Ciprofloxacin (10 mg kg^−1^, 30.18 µmol L^−1^) was used as a positive control. d–f) Histological scores of mice ileums (d), cecums (e), and colon (f), respectively, after different treatments for 7 days. g) FCLAPs effectively rescued mice suffering from intestinal inflammation caused by *E. coli* O157:H7. Hematoxylin and eosin (H&E) staining images (magnification × 100) of ileums, cecums, and colons in different groups after treatment for 7 days. A scale bar represents 200 µm. All images are representative of three independent observations. h) Food surface decontamination model. Treatments with 2 mL of KTA (50 µg mL^−1^), KTR (100 µg mL^−1^), RI (150 µg mL^−1^), and RT (100 µg mL^−1^) for 10 min dramatically reduced bacterial number of *E. coli* O157:H7 on the contaminated food surfaces. The surfaces of cherry tomatoes were inoculated with *E. coli* O157:H7 (around 6.20 log CFU g^−1^) for 1 h before treatments. ND: not detected. The limit of detection was 1.0 log CFU g^−1^. For cultures in which colony counts were less than the limit of detection, statistical analysis was performed with values set at the limit of detection. Data presented as mean ± SD, *n* = 3. *P*‐values in (d–f) were calculated using unpaired Student's t‐test. *P*‐values in (h) were calculated using one‐way ANOVA.

Next, we asked whether the FCLAPs exhibit therapeutic efficacy in infectious models. To this end, we constructed gut colonization models of an intestinal pathogen in mice, where the intragastric dose of *E. coli* O157:H7 (2 × 10^8^ cells) was colonized into the gastrointestinal tract. At 24 h post‐infection, mice were treated with either PBS (negative control), the conventional antibiotic ciprofloxacin (positive control), or FCLAPs, once daily for seven consecutive days (**Figure** **3**a). After administration, we quantified the bacterial count in feces and intestinal segments of mice in each treated group. The strain was able to colonize mice on the second day post‐infection to the ninth day, with a peak of colonization observed on the fifth day. In the PBS‐treated group, colonization declined from the fifth day to the end of the experiment, probably in response to immune system action.^[^ ^23^ ^]^ FCLAPs exerted potent antimicrobial efficacy, resulting in an almost 4.5‐log CFU reduction compared with the negative control group (*P* < 0.0001) (Figure 3b). Remarkably, a substantial decrease in bacterial burden was observed in the FCLAPs‐treated group on the 3rd day post‐colonization. As expected, the reduction of the fecal number of *E. coli* was consistent with the decrease of colonized bacteria in the gut (Figure 3c).

*E. coli* O157:H7, a well‐known foodborne pathogen, has been responsible for a total number of 269 foodborne disease outbreaks, resulting in more than 4000 illnesses in the United States during 1998–2019.^[^ ^24^ ^]^ Meanwhile, as an enteric pathogen, it is the leading cause of intestinal infections such as intestinal epithelial barrier disruptions, acute enteritis, and intestinal hemorrhagic colitis.^[^ ^25^ ^]^ In light of its high morbidity and the potential damage to the intestine, we further examined the physiological status of *E. coli*‐colonized mice treated with FCLAPs from a histological viewpoint. The H&E images were captured and the histopathological injury was scored (Figure 3d–f). We observed that the untreated, and PBS‐treated groups showed typical characteristics of ruptured epithelial cells, decreased crypt depth, and disrupted lamina propria and mucous membrane, in which inflammatory cell infiltration appeared (Figure 3d; Figure S7, Supporting Information). In sharp contrast, mice savaged intragastrically with FCLAPs exhibited more moderate tissue damage, with structurally intact intestinal epithelial cells and relatively normal crypt structure. KTA (9.87 µmol L^−1^), KTR (18.95 µmol L^−1^), RI (38.31 µmol L^−1^), and ciprofloxacin (30.18 µmol L^−1^) were essentially equipotent in attenuating the colitis caused by *E. coli* infection, whereas RT (24.81 µmol L^−1^) was slightly less active than them. Furthermore, we observed a striking decrease in the KTA‐treated group regarding the tissue damage scores in comparison with the untreated one (Figure 3g; Figure S7, Supporting Information). According to the literature, AMPs enhance host cells’ innate immunity to fight against the bacteria residing inside the intestine.^[^ ^2^, ^26^ ^]^ Our results supported a remarkably decreased inflammation in the intestine of mice treated with FCLAPs, which further added to evidence that the bacterial burden in the *E. coli*‐colonized mice model has effectively reduced. These data demonstrate that KTA and KTR might be promising alternatives to antibiotics for the treatment of *E. coli* O157:H7 infection.

Given the attractive pharmacological efficacy of FCLAPs in vivo, we further investigated their surface decontamination capability on fresh food models. The surfaces of cherry tomatoes were inoculated with *E. coli* O157:H7 (around 6.20 log CFU g^−1^) before treatment. The four AMPs dramatically decreased the number of *E. coli* O157:H7 on the surface of contaminated cherry tomatoes in 10 min (Figure 3h). Noteworthily, a complete reduction of surface‐attached *E. coli* was observed in the KTA‐treated group (50 µg mL^−1^), with no detectable bacteria (limit of detection was 1.0 log CFU g^−1^). Hence, these peptides are applicable to food matrices as disinfection agents for averting the potentially detrimental effect of foodborne pathogenic infections. Considering the results of antibacterial activity both in vivo and in vitro, KTA and KTR were selected for subsequent mechanistic investigations.

### 2.4 FCLAPs Disrupt Bacterial Morphology and Integrity

**Figure 4:**
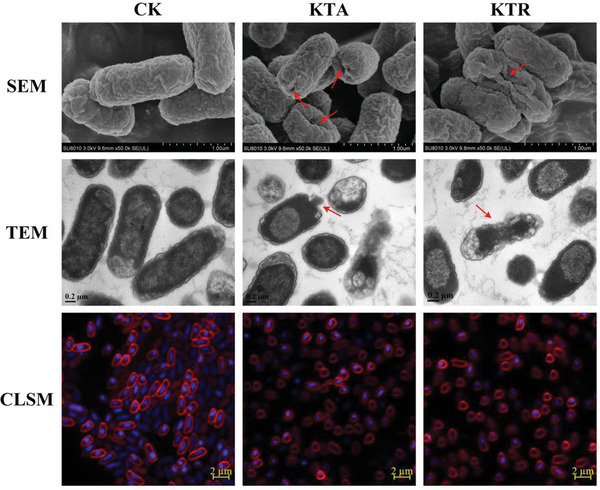
Morphological studies of *E. coli* O157:H7 before and after treatment with KTA and KTR. Representative images of scanning electron microscopy (upper), transmission electron microscopy (middle), and confocal laser scanning microscopy analysis (lower). *E. coli* O157:H7 cells were treated with 4 × MIC KTA and KTR for 1 h before observation, respectively. For SEM images, a scale bar represents 1.0 µm. For TEM images, a scale bar represents 0.2 µm. For CLSM images, the *E. coli* O157:H7 cell membranes and nucleoids were stained with FM4‐64 (red) and DAPI (blue), respectively. A scale bar in the CLSM image represents 2.0 µm. Regions of interest are indicated by red arrows. All images are representative of three biologically independent experiments performed with similar results.

To directly visualize the effect of the FCLAP treatments on bacterial cells, we performed a range of microscopical analyses using *E. coli* O157:H7 ATCC 35150 as a model. First, scanning electron microscopy (SEM) and transmission eletron microscopy (TEM) images illustrated the surface morphological and ultrastructural changes of *E. coli* cells upon FCLAP treatments, respectively, as given in **Figure** **4**. Untreated cells displayed a normal appearance with a characteristic rod‐like shape and intact membrane structure. Upon treatment with KTA, bacterial cells presented visible pore formations and shrunken cell membranes. Moreover, many KTR‐treated cells appeared shriveled as raisins and exhibited indents on the cellular surface, which could result from the collapse of the bacteria and loss of cellular contents. When bacterial cells were exposed to RI, most of them exhibited contraction at division sites, however, some protrusions similar to blebs were observed at the mid‐section of bacteria cells upon treatment with RT (Figure S8, Supporting Information). These alterations could be explained by the literature which reveals that the outer membrane is less stably connected to the cell wall at sites of cell division, whereby midcell blebs or constriction usually occurred.^[^ ^27^ ^]^ In addition, localized swelling or bulges that encircled the entire circumference of the bacterial cells were also observed upon treatment with RI and RT. Such morphological responses might be attributed to the disruption of peptidoglycan synthesis, especially the inhibition of penicillin‐binding proteins (PBP) under stress conditions.^[^ ^28^ ^]^

Further, confocal laser scanning microscopy (CLSM) imaging was used to garner more information about the morphological alterations of bacteria upon FCLAP treatments. *E. coli* cells were stained with two dyes that report on membrane morphology (FM4‐64) and nucleoid morphology (DAPI) and imaged at high resolution.^[^ ^29^ ^]^ Decreases in cell size (both rod diameter and length) have been observed in the *E. coli* cells treated with KTA and KTR as compared to the untreated samples (Figure 4). Also, KTA and KTR caused stronger effects on bacteria morphology than RI and RT, which corresponded to their antibacterial activities (Figure S9, Supporting Information). This change in bacterial morphology has generally termed the formation of “ovoid cells”, in which the bacterial rods have decreased in length and become oval or round shaped upon antibacterial treatment. Previous studies demonstrated that ovoid cell formation always occurs following inhibition or disruption of peptidoglycan synthesis by antimicrobial agents.^[^ ^30^ ^]^ Thus, this observation hints that PGN might serve as an important antibacterial target for FCLAPs.

**Figure 5:**
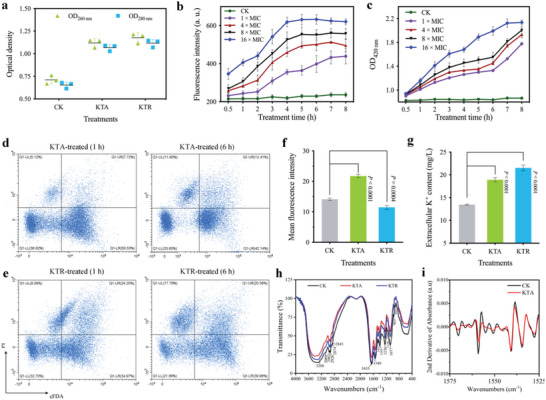
KTA and KTR exert bactericidal effects through membrane‐mediated mechanisms. a) The count of nucleic acids and protein leakage in *E. coli* O157:H7 culture medium determined after treatment with 4 × MIC FCLAPs for 1 h. b) Dynamic curves of the permeability of outer membrane probed with NPN. *E. coli* cells were treated with KTA at 1 ×, 4 ×, 8 ×, and 16 × MIC, respectively. c) Dynamic curves of the inner membrane permeability, determined optically at 420 nm by measuring the release of cytoplasmic *β*‐galactosidase. Bacterial cells were incubated with ONPG in the presence of 1 ×, 4 ×, 8 ×, or 16 × MIC KTA. d,e) Flow cytometry dot plots of KTA‐treated (d) or KTR‐treated (e) *E. coli* O157:H7 which were stained by cFDA (*x*‐axis) and PI (*y*‐axis). Bacterial cells were sampled after 1 and 6 h treatment, respectively. f), Membrane potential measured by fluorescence of Rhodamine 123 after treatments of FCLAPs for 1 h. g) Content of K^+^ leakage from *E. coli* after 1 h treatment of FCLAPs at a concentration of 4 × MIC was analyzed by atomic absorption spectrometry. h,i) FTIR spectra (4000–400 cm^−1^ wavenumber) (h) and the second derivative of the absorbance (1575–1525 cm^−1^) (i) of *E. coli* O157:H7 treated with 4 × MIC KTA for 1 h. The spectrum derived from untreated sample was labeled as control (CK). All experiments were performed as three biologically independent experiments, data presented as mean ± SD, *n* = 3. *P*‐values in (f,g) were calculated using unpaired Student's *t*‐test.

The ability of FCLAPs to induce efflux of intracellular components was studied, as it characterizes their effects on bacterial cell integrity.^[^ ^31^ ^]^ A significantly larger amount (*P* < 0.05) of interior substances, including nucleic acids and proteins, were released from bacterial cells upon AMPs treatments (**Figure** **5**a). The results were in line with the CLSM observations, in which the nucleoid area was shrunk and DNA count was decreased as seen by DAPI staining (Figure 4).

### 2.5 Multi‐Omics Investigation of Antibacterial Molecular Mechanism

**Figure 6:**
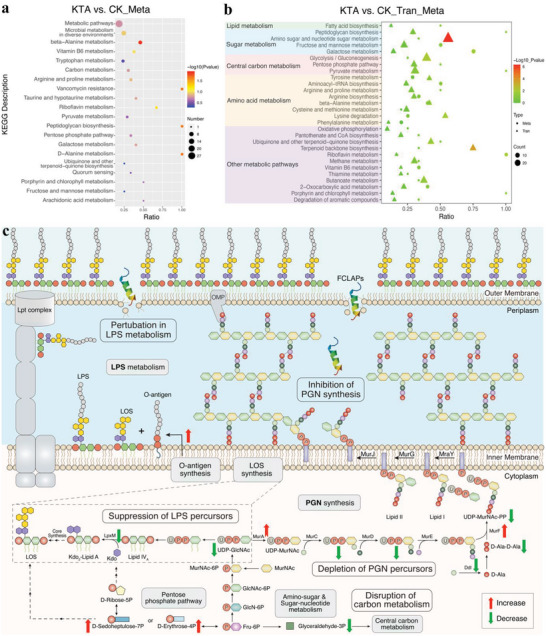
Metabolic perturbation of *E. coli* treated with KTA. a) KEGG Pathway enrichment analysis of differential metabolites between KTA‐treated and control group. Significantly enriched pathways were selected for plotting (*P* < 0.05). b) KEGG Pathway enrichment analysis of integrated transcriptomic and metabolomics data between *E. coli* O157:H7 treated with KTA and untreated cells. Significantly enriched pathways were selected for plotting (*P* < 0.05). c) Schematic diagram summarizing the main antibacterial mechanisms of KTA in combating *E. coli* O157:H7. The most significant findings on the inactivation mechanism of KTA include inhibition of LPS and PGN metabolism, depletion of key precursors for LPS and PGN biosynthesis, and perturbation of central carbon metabolism and energy metabolism.

The encouraging in vitro and in vivo antimicrobial activities, as well as the disruptive action on bacterial cells of KTA and KTR, inspired us to further dissect their underlying mechanistic information. Considering that bacteria always initiate molecular responses to maintain cellular homeostasis when countering the perturbance of antibacterial substances, we employed large‐scale omics approaches to study the antibacterial mechanism of FCLAPs. First, we used RNA Sequencing (RNA‐Seq) technology to profile the transcriptomic responses of *E. coli* O157:H7 and specific gene sets modulated by the treatments. Furthermore, the RT‐qPCR verification results showed consistency with the RNA‐Seq data, indicating the reliability of the transcriptomic profiles (Figures S10 and S11, Supporting Information). Untargeted metabolomics was applied to characterize the metabolic changes in *E. coli* treated with FCLAPs at 1 h. KEGG pathway analysis revealed a significant enrichment of differentially regulated metabolites involved in fatty acids metabolism, peptidoglycan biosynthesis, amino acid metabolism, and energy metabolism (**Figure** **6**a; Figure S12, Supporting Information).

To decipher the joint gene‐metabolite pathways, we profiled the response of *E. coli* post FCLAP exposures using a systematically integrated transcriptomic and metabolomics approach. The top significantly enriched metabolic pathways with integrated genes were described in Figure 6b; Figure S12 and Table S5, Supporting Information. On the whole, as illustrated in Figure 6c, the most significant findings on the inactivation mechanism in the multi‐omics study include 1) suppression of bacterial membrane lipid metabolism via multiple targets (i.e., fatty acid, phospholipids, peptidoglycan, and LPS); 2) perturbation of key precursors of membrane components synthesis (amino‐sugar and sugar‐nucleotide metabolism, etc.); and 3) disruption of central carbon metabolism and the downstream metabolism of energy and amino acids.

In terms of the impact on membrane lipid metabolism, treatment with FCLAPs significantly up‐regulated the transcriptional level of genes associated with fatty acid degradation (*fad*E, *fad*A, *fad*B, *fad*I, etc.), glycerolipid/glycerophospholipid metabolism (*cls*B, *cls*C, *glp*D, etc.), and lipopolysaccharide biosynthesis (*pag*P, *ept*A, etc.). Meanwhile, the levels of two well‐known metabolites in bacterial fatty acid metabolism, namely, palmitoleic acid, and arachidonic acid, were significantly increased. A number of phospholipids, including PG (16:1/17:1), PG (8:0/17:1), PE (17:0/18:2), PE (18:0/19:2), LPE 18:3, LPE 20:4, etc., underwent a remarkable increment in their levels after FCLAP treatments (Table S5, Supporting Information). The results suggest that the FCLAPs markedly compromised the main metabolites of bacterial membrane lipids, especially those related to the biosynthesis of outer and inner membrane components.

The impacts of FCLAPs on amino‐sugar and sugar‐nucleotide metabolism were also remarkable. Both pathways are fundamental pools that feed key precursors into the biosynthesis of PGN and LPS. Notably, there was a significant enrichment of genes and metabolite features involved in PGN biosynthesis including *mur*A, *mur*F, *dac*C, *mrc*A, and *mrc*B genes accompanied by downregulation of metabolite features of D‐Alanyl‐D‐alanine. This effect might be ascribed to the remarkable perturbation in the levels of amino sugar and nucleotide sugar intermediates that provide precursors for peptidoglycan biosynthesis. O‐antigen is a crucial building block that forms the outer core of the LPS layer in Gram‐negative microbes. Here, the level of fourteen key genes involved in O‐antigen nucleotide sugar biosynthesis was significantly increased following treatments, indicating that the bacteria might bear impaired LPS components in response to the FCLAPs.

Treatments with FCLAPs also remarkably disturbed the central carbon metabolism, including glycolysis/gluconeogenesis, pentose phosphate pathway (PPP), and citrate cycle (TCA cycle). Noteworthily, KTA and KTR treatments induced a profound increment in the concentration of a PPP metabolite, D‐erythrose 4‐phosphate, a precursor that is crucial for the LPS biosynthetic pathway.^[^ ^32^ ^]^ So far as we know, the cellular response to AMPs has not been studied at transcriptional and global metabolic levels. Our results are greatly conducive to building a first complete picture of the metabolic changes, shedding light on the multimodal mechanisms involved in the antibacterial action of the FCLAPs. Overall, a possible inactivation mechanism of FCLAPs is strongly related to their membrane‐targeting activity and concomitant destructive effect on membrane lipid metabolism. To this end, we performed a set of experiments to characterize the distinctive features of FCLAPs as membrane‐disruptive antimicrobials.

### 2.6 FCLAPs Act as Membrane‐Disruptive Antimicrobials

To determine how FCLAPs affect the cell membranes of living bacteria, we conducted a panel of experiments to characterize the changes in membrane properties upon *E. coli* O157:H7 treated with FCLAPs. First, insights into the mode of bactericidal action of FCLAPs were obtained by the flow cytometric analysis. We evaluated whether the peptides compromised membrane integrity using the membrane‐impermeable DNA stain propidium iodide (PI), which cannot enter intact cells. There were no considerable changes in the PI‐stained population within 1 h of peptides exposure, which might be ascribed to the peptides merely destabilizing the outer membrane without rupturing the inner membrane. Considering the existence of the outer membrane, the cytoplasmic membrane was not severely affected at that time. As the exposure time prolonged, a rapid decrease of live cells was spotted after KTA and KTR treatments. By 6 h, the percentages of PI‐positive cells significantly increased with more severe damage to the cytoplasmic membrane, suggesting that the peptides permeabilized both the outer and inner membrane (Figure 5d,e; Figure S13, Supporting Information). These results further confirmed that FCLAPs could kill bacteria by causing cell membrane rupture, indicating strong membrane‐permeabilizing ability with time‐dependent effects. Meanwhile, the intracellular enzymatic activity was determined by carboxyfluorescein diacetate (cFDA), a non‐fluorescent that diffuses readily across the cell membrane and is converted by the esterase into a green florescent metabolite (cF).^[^ ^33^ ^]^ The percentages of cF‐stained cells decreased in a time‐dependent manner, suggesting the perturbation of esterase activity and inhibition of metabolic performance during the FCLAP treatments.

Membrane permeability analysis was then employed to characterize the features of peptide‐membrane interaction. The capability of FCLAPs to permeabilize the outer membrane was assessed by an NPN uptake assay, according to our previous study.^[^ ^34^ ^]^ Concretely, NPN is repelled from the OM and cannot penetrate the OM since the negative charge, whereas its permeabilization can occur through a damaged outer membrane. The results demonstrated that FCLAP treatments could directly contribute to outer membrane permeabilization in a time‐ and concentration‐dependent manner (Figure 5b; Figure S14, Supporting Information). The kinetic data of maximum NPN uptake values were saturated at around 6 h when cells were treated with KTA at 4 × MIC, while at 16 × MIC, the duration to reach saturation was shortened to 4 h. As for the inner membrane, when it is compromised, the cytoplasmic *β*‐galactosidase could release from the cytoplasm and hydrolyze ONPG into yellow ONP. Hence, the alteration of IM permeability was assessed by monitoring the ONP production.^[^ ^35^ ^]^ It was noticed that there was a low increase in inner membrane permeability within the first 6 h of treatment (Figure 5c; Figure S14, Supporting Information). Afterward, the permeability started to increase, suggesting that the alterations on the inner membrane gradually became severe. As treatment time was prolonged to 6 h, the occurrence of permeabilization on both the outer and inner membranes appeared to be synchronized over time.

To further demonstrate how the *E. coli* cells respond to the exposure of FCLAPs, the membrane potential upon treatment was detected.^[^ ^36^ ^]^ Results showed that the KTA could contribute to significant (*P* < 0.0001) hyper‐polarization of membrane potential, whereas the depolarized bacterial membranes were observed in the presence of KTR (Figure 5f). According to the literature, the great perturbance in membrane potential was found to be coupled with the increment of membrane permeability and anomalous ion fluxes.^[^ ^37^ ^]^ Thereby, we next used atomic emission spectroscopy to quantify the concentration of outflow K^+^ post FCLAP treatments. As depicted in Figure 5g, the extracellular K^+^ content significantly increased from 13.47 to 18.92 and 21.53 µg mL^−1^ in response to KTA and KTR, respectively. In our investigation, levels of disturbance on the membrane potential were in correlation with the increase of membrane permeability, loss of cell viability, and efflux of K^+^, therefore, providing a deeper understanding of the microbial behaviors upon the FCLAPs exposure.

Fourier transform Infra‐Red (FTIR) spectra were conducted to explore changes in structural conformation of the cell membrane following FCLAP treatments. Results showed that FCLAPs inflicted major conformational changes in cell envelope functional units, specified as a general reduction of peak intensities relative to the control group (Figure 5h). Among the FCLAPs, KTA exhibited the most severe damage to the conformation of membrane components including saturated lipids at 2926 (CH_2_ antisymmetric stretching) and 1395 cm^−1^ (‐CH_3_ umbrella symmetric bending vibration), amide bands at 1655 (amide I) and 1540 cm^−1^ (amide II) and saccharide bands at 1236 cm^−1^ (C—O stretching vibration), as well as 963 cm^−1^ (ring of saccharide) (Figure 5h,i; Figure S15, Supporting Information). Note that there was a substantial reduction of the 1655 (amide I) and 1560 cm^−1^ (C—N asymmetric stretching vibration) peaks, indicating the weakening of glucosamine phosphates moiety in the lipid A upon KTA exposure. The LPS targeting also echoed the outer membrane disarrays that were seen in membrane morphology and permeability changes. It was also worth mentioning that changes in the PGN were observed in cells treated with KTA and RI (Figure S15, Supporting Information). We detected noticeable decreases in the 1645 cm^−1^ amide I and 1540 cm^−1^ amide II in the KTA‐treated samples when compared to untreated control, indicating damage to the amide groups of PGN. We also observed the reduction of the saccharide bands (1166, 1077, and 963 cm^−1^) that form the sugar units, suggesting the disintegration of the glycan strands following KTA and RI treatment.^[^ ^38^ ^]^ The impairment of the PGN structure could be one of the reasons for the formation of “ovoid cells” seen in CLSM images (Figure 4). Based on the spectral changes, FCLAPs were indicated to initiate membrane damage by targeting multiple cell envelope units, especially LPS and PGN. Up to this stage, we have confirmed the distinct membrane‐targeting properties of FCLAPs and elaborated on how their exposures alter and disintegrate cell membrane components.

### 2.7 FCLAPs Function via a Dual‐Targeting Mechanism

**Figure 7:**
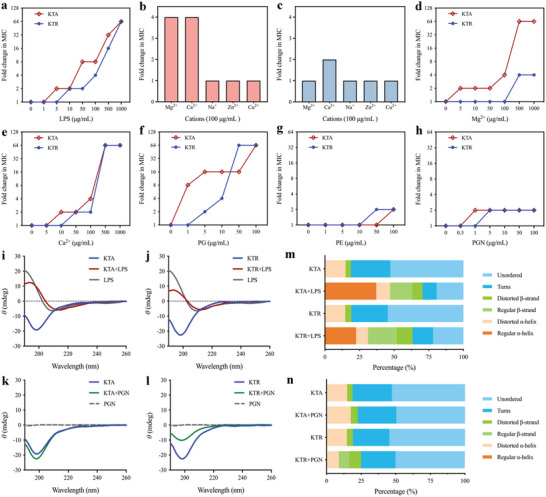
KTA and KTR exert antibacterial effects through peptide‐membrane interaction. a) Exogenous addition of LPS isolated from *E. coli* O155:B5 impairs the antibacterial activities of KTA and KTR against *E. coli* O157:H7 in a dose‐dependent manner, determined by chequerboard broth microdilution tests. b,c) The MIC changes upon exogenous addition of different cations (100 µg mL^−1^ of Mg^2+^, Ca^2+^, Na^+^, Zn^2+^, Cu^2+^) to KTA and KTR, respectively. d,e) The MIC changes of KTA and KTR against *E. coli* O157:H7 in the presence of Mg^2+^ and Ca^2+^. f–h) Exogenous addition of PG, PE, and PGN, respectively, impairs the antibacterial activities of KTA and KTR against *E. coli* O157:H7 in a dose‐dependent manner, determined by chequerboard broth microdilution tests. i,j) Normalized CD spectra of KTA and KTR (0.1 mmol L^−1^) in the presence and absence of LPS (25 µmol L^−1^). k,l) Normalized CD spectra of KTA and KTR (0.1 mmol L^−1^) in the presence or absence of PGN (1 mmol L^−1^). m,n) Abundance ratio of secondary structure motifs analyzed from CD spectral data.

Given that FCLAPs have been asserted to target the bacterial membrane, we next focused on identifying the interaction sites between the peptides with bacterial cell membranes. FCLAPs are large molecules (2025 and 2110 Da for KTA and KTR, respectively), whereas the cut‐off for compounds to permeate the outer membrane is around 600 Da.^[^ ^1a^ ^]^ We therefore considered that before permeating the bacterial membrane, FCLAPs might initially target the outermost layer of the cell envelopes. Since lipopolysaccharides (LPS) are an abundant component that is present in the outer leaflet of the Gram‐negative bacterial cell membrane, we first evaluated the LPS‐membrane interaction via a competitive inhibition assay with purified LPS of *E. coli* origin. The results revealed that adding exogenously LPS dramatically suppressed the activity of FCLAPs in a dose‐dependent manner (**Figure** **7**a; Figure S16a, Supporting Information). Especially, the antibacterial activity of KTA was largely abolished by the addition of LPS as compared with the rest of the peptides, suggesting the strongest LPS binding activity among all FCLAPs. This result could explain the robust antibacterial activities of KTA and KTR, as it is reported that the bactericidal potential of cationic AMPs correlates well with their binding capacity to LPS.^[^ ^2a^ ^]^ The negatively charged phosphates on lipid A in LPS can be modified to either neutral or positively charged by divalent cations, which confers resistance to cationic AMPs.^[^ ^28^, ^39^ ^]^ Therefore, we further investigated whether these AMPs bound to the negative domains of LPS by adding Ca^2+^ and Mg^2+^ (Figure 7b–e; Figure S16b–e, Supporting Information). Results in the cation binding assays revealed that these two cations can suppress the antibacterial activity of FCLAPs, especially KTA, with 64‐folds change in MIC under a high level of cations’ addition (> 500 µg mL^−1^).

To better understand the binding of FCLAPs to LPS, the global conformational discrepancies between peptide in free solution and in complex with LPS was assessed using CD spectra.^[^ ^40^ ^]^ All FCLAPs indicated a random coil nature in an aqueous solution, while conformational changes were observed with a positive maximum at 195 nm and a negative band at 208 to 220 nm, suggesting predominantly helical conformations (Figure 7i,j; Figure S16i,j, Supporting Information). Calculating the CD component bands by the CONTIN method, we found that the helical contents for KTA and KTR were increased by 32.2% and 16.4%, respectively, in the presence of LPS (Figure 7m). These results indicated a strong electrostatic interaction between the positively charged residues in FCLAPs and negatively charged LPS that induced a conformational transition of the peptides into helical states.

Once destabilize the LPS leaflet, the peptides would then reach and attach to the inner monolayer of the outer membrane, namely the phospholipid leaflet, to exert their antibacterial activities. To investigate whether FCLAPs subsequently disrupted the phospholipid monolayer of the outer membrane, we assessed the exogenous addition of two representative phospholipids (PE and PG). The results showed that PG and PE effectively suppressed the antibacterial effect of FCLAPs in a dose‐dependent manner (Figure 7f–g; Figure S16i,j, Supporting Information), reflecting a disturbance of the peptides against the inner surface of the outer membrane. The activities of FCLAPs towards both LPS and phospholipids would serve to explain the prominent increase in outer membrane permeability (Figure 5b; Figure S14, Supporting Information). In turn, permeabilizing the membrane could also promote the attachment of FCLAPs to the PGN layer.

Afterward, we speculated that the paralysis of the outer membrane would facilitate the passage of the FCLAPs span the outer membrane into the PGN target. To investigate this hypothesis, we set out to elucidate the FCLAPs‐PGN interaction mechanism. As shown in Figure 7h, low levels of PGN had a neglectful influence on the antibacterial potency of either KTA or KTR, while the antibacterial efficacies were found to be slightly inhibited with the increase in PGN concentration. We therefore studied the direct interaction of FCLAPs with PGN using CD spectroscopy (Figure 7k,l; Figure S16k,l, Supporting Information). Despite the physical presence of the outer membrane might somehow weaken the peptide‐PGN interaction, changes in the secondary structure components of the peptides were observed upon the addition of exogenous PGN (Figure 7n; Figure S16n, Supporting Information). The results provided evidence for our speculation that PGN acts as an additional antibacterial target of the peptides. Collectively, we propose a unique dual‐targeting mechanism in that FCLAPs initially cause the destabilization of LPS through ionic interactions, thus subsequently allowing the uptake and intercalation of peptides into the PGN layer. Since the mechanism by which AMPs can reorient in and disrupt the PGN after outer membrane destabilization has not been reported, it is likely a peculiar feature of FCLAPs as membrane‐disruptive antimicrobials.

### 2.8 FCLAPs Target PGN by Blocking its Synthesis

**Figure 8:**
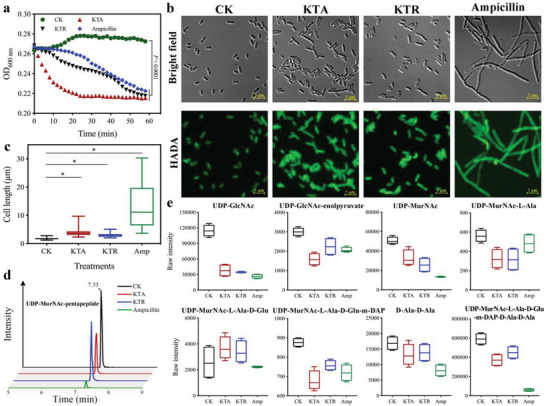
KTA and KTR target peptidoglycan by blocking its synthesis. a) Peptidoglycan integrity of the log‐phase *E. coli* grown in the absence of the presence of 0.625 µmol L^−1^ KTA (1/4 × MIC), 1.18 µmol L^−1^ KTR (1/4 × MIC), or 2.86 µmol L^−1^ ampicillin (1/4 × MIC). Ampicillin was used as a positive control. The optical density at 600 nm (OD600 nm) was determined as an indication of peptidoglycan degradation. Data recorded at 60 min were analyzed using an unpaired Students’ *t*‐test. b) Peptidoglycan morphology of the log‐phase *E. coli* grown in the absence or presence of 0.625 µmol L^−1^ KTA (1/4 × MIC), 1.18 µmol L^−1^ KTR (1/4 × MIC), or 2.86 µmol L^−1^ ampicillin (1/4 × MIC). Ampicillin was used as a positive control. Untreated or treated cells were incubated with HADA for 30 min, followed by observation using confocal laser microscopy. Scale bar is 5 µm. Images are representative of *n* = 3 separate experiments with 10–20 images acquired for each treatment group in each experiment. c) Comparison of individual cell length. A total of 30 measurements was collected each for cell length per treatment. Data are representative of 3 independent experiments. Results are represented as a box‐whisker plot with data represented from minimum to maximum. Data were analyzed using an unpaired Students’ *t*‐test. * indicates *P* < 0.0001. d) Intracellular accumulation of the peptidoglycan precursor UDP‐MurNAc‐pentapeptide of *E. coli* upon treatment with 0.625 µmol L^−1^ KTA or 1.18 µmol L^−1^ KTR as shown in the extracted ion chromatograms of LCMS analysis. Untreated and ampicillin‐treated (2.86 µmol L^−1^) cells were used as controls. UDP‐MurNAc‐pentapeptide was identified by mass spectrometry at *m/z* 1192.3329 eluting at 7.33 min. The figure is representative of 4 independent experiments. e) Box‐whisker plots for significantly perturbed precursors of peptidoglycan biosynthesis following treatment with 0.625 µmol L^−1^ KTA (1/4 × MIC), 1.18 µmol L^−1^ KTR (1/4 × MIC), or 2.86 µmol L^−1^ ampicillin (1/4 × MIC). Amp, ampicillin. *n* = 4 independent experiments from which data was collected. Results are displayed as box‐whisker plots with data represented from minimum to maximum.

Given the microscopy observations, multi‐omics analysis, and peptide‐membrane interaction assays alerted us to a second potential target of FCLAPs, the peptidoglycan, we next sought to explore their PGN‐disruptive effects. To gain clues about this potential target, we revisited our CLSM images of KTA or KTR‐treated *E. coli* cells (Figure 4). We observed distinct changes in bacterial shape and size, indicating the disruption of *E. coli* peptidoglycan metabolism upon treatments with high concentrations (4 × MIC) of FCLAPs. Next, to further dissect the effect of FCLAPs on the PGN layer, we assessed the PGN integrity by measuring the susceptibility of KTA or KTR‐treated cells to lysozyme. As depicted in **Figure** **8**a, the OD_600 nm_ of KTA‐treated cells was reduced by 17.2% within 20 min incubation time, suggesting a rapid lytic and killing activity of this AMP. Ampicillin was employed as a positive control as it is a cell wall‐active antibiotic that blocks the final cross‐linking step, causing a loss of integrity and bacterial lysis.^[^ ^41^ ^]^ Based on the Students’ *t*‐test analysis, the differences between PGN susceptibilities of KTA or KTR‐treated and untreated cells at the 1‐h time point were significantly different (*P* < 0.0001), while there was statistically insignificant difference between those of KTA‐ and ampicillin‐treated cells. These results support our hypothesis that FCLAPs compromised the integrity of PGN, contributing to PGN degradation and cell lysis.

Next, we set to get a glance at cell morphology changes to understand whether KTA and KTR inhibited PGN biosynthesis. To this end, we applied a published protocol for in situ labeling of PGN with 7‐hydroxycoumarin‐3‐carboxylic acid 3‐amino‐D‐alanine (HADA), a fluorescent‐tagged D‐amino acid that incorporates actively into the terminus of the stem peptide of growing PGN.^[^ ^42^ ^]^ By using CLSM, we examined the phenotype of *E. coli* cells grown in sub‐MIC levels of FCLAPs (Figure 8b). At 0.25 × MIC, marked effects of KTA and KTR on PGN morphology were detected by the aberrant cell elongations, the absence of septal structure, and even the collapse of cell walls. Such phenotype has been generally described as “filamentation”. Bacteria with a characteristic filamentation appearance were presumed to be cells whose elongation‐division cycles have been disrupted under adverse environmental conditions.^[^ ^30^ ^]^ According to the literature, antibacterial agents that inhibit the synthesis of PGN usually induce cessation of septum formation, resulting in cell elongation without division.^[^ ^43^ ^]^ It was also noted that KTA and KTR induced comparable morphological changes in cells with those by treatment with the PGN synthesis inhibitor ampicillin (Figure 8b,c). Combined, we reasoned that KTA and KTR would repress the septal wall synthesis, block PGN cleavage at the division septa, and thus generate the abnormally long filaments.

To investigate which stage of PGN biosynthesis is interrupted by KTA and KTR, we measured the fundamental building blocks on the synthesis pathway. Notably, PGN was substantially perturbed by KTA and KTR, wherein the abundances of seven PGN biosynthetic precursor metabolites were considerably decreased, including UDP‐GlcNAc, UDP‐MurNAc, UDP‐MurNAc‐L‐Ala, and UDP‐MurNAc‐L‐Ala‐D‐Glu‐m‐DAP, etc. (Figure 8e). Among the core precursors, we focused on assessing the build‐up of the final cytoplasmic intermediate, UDP‐MurNAc‐pentapeptide (Figure S17, Supporting Information).^[^ ^41^, ^44^ ^]^ Similar to the perturbation seen in *E. coli* treated with ampicillin, albeit to a lesser extent, the concentration of UDP‐MurNAc‐pentapeptide was strikingly declined in the presence of KTA and KTR (Figure 8d). These suggested that KTA and KTR strongly inhibited the metabolism of peptidoglycan by blocking the membrane‐associated steps of its biosynthesis. We therefore verified peptidoglycan metabolism as another potential target of KTA and KTR, which to our knowledge is distinct from that of any previously reported cationic AMP.

## 3 Discussion

The dearth of drug candidates against Gram‐negative bacteria, which is largely ascribed to the distinctive cell envelope and extensive drug resistance of the microbes, remains a key concern in the healthcare and food processing sectors.^[^ ^45^ ^]^ AMPs have been explored as promising antibiotic alternatives to address the formidable challenge of the shortage of drug candidates and alarming antibiotic resistance.^[^ ^46^ ^]^ However, the high cytotoxicity and low in vivo efficacy impeded the application of AMPs in clinical and food settings. In this regard, practical strategies to improve the performance of AMPs and their success rate in clinical trials have been summarized in previous systematic reviews.^[^ ^10^ ^]^ Herein, we reported AMPs modified by mutating amino acid residues of a natural food‐derived AMP–Leg2. Benefiting from the optimized hydrophobicity, or redesigned net charge, FCLAPs exhibited orders of magnitude more effective against a variety of Gram‐negative bacterial pathogens, as compared with the parental AMP (Table 1). More importantly, KTA and KTR effectively kill the foodborne pathogenic *E. coli* O157:H7 both in vitro and in vivo, with activities comparable to or higher than the conventional antibiotics (Table 1 and Figure 3). Especially, their robust efficacies in food surface decontamination brighten the prospects of the peptides in averting the potential detrimental effect caused by foodborne pathogenic infection. FCLAPs also displayed low cytotoxicity to mammalian cells, good biocompatibility, and high proteolytic resistance (Figure 2c–f and Figure 1e,f). Furthermore, we found that FCLAPs induced much lower drug resistance than colistin when used in sub‐inhibitory concentration (Figure 2b). Together, we demonstrated that KTA and KTR are exceptionally effective in combating *E. coli* O157:H7 and have great potential as a novel class of antimicrobial options.

Inspired by the satisfactory antibacterial performance, we next attempt to explain the outstanding antibacterial activity of KTA and KTR by analyzing their composition and sequence motifs. More recently, studies on the structure‐activity relationship have led to the development of novel peptide analogs with better antimicrobial and therapeutic potentials by altering certain physicochemical and structural properties, involving net charge, hydrophobicity, and sequence length.^[^ ^47^ ^]^ As seen in the structure prediction and analyses (Figure 1b–d), coupled with the ideal number of positive charges and appropriate side‐chain hydrophobicity, KTA and KTR presented appreciated antimicrobial performance. The calculated percentage of secondary structure components for KTA was around 98% *α*‐helix in membrane‐mimetic environment (Figure S2, Supporting Information), indicating that KTA possessed a strong propensity for an *α*‐helical conformation in the presence of membrane lipids. It is reported that the stable *α*‐secondary structure facilitates the insertion of the peptides into the cell membrane, and hence makes a significant contribution to improving antimicrobial efficacies.^[^ ^48^ ^]^ In addition, the arginine‐rich *α*‐helix in the middle part of KTA might endow it with excellent antibacterial activity as well as LPS‐binding and neutralization capacity compared with Leg2. Previous studies indicated that the cationic residues are essential in the LPS binding motif for membrane permeabilization.^[^ ^9^ ^]^ Given that KTR has the strongest positive charge due to the presence of four arginine, it was not surprising that this cationic region is capable of promoting the interaction of AMPs with anionic LPS and bacterial membranes. Also, the hydrophobicity of KTR was lower than other three peptides, which may be better to bind LPS. However, the antibacterial effects of RI and RT were unsatisfactory as compared with KTA and KTR (Table 1; Table S1, Supporting Information). It was found that an excessive decrease of sequence length could not enhance the antimicrobial activity of them as expected. Here we concluded that the physiochemical properties of the AMPs, involving the propensity of *α*‐helical conformation, contents of cationic residues, hydrophobicity feature, and sequence length, are related to the antibacterial performance by influencing the LPS‐binding efficiency. The knowledge gleaned here may inspire the modification of novel AMPs.

We further described an integrated multi‐omics approach to characterize the molecular mechanisms of the FCLAPs against *E. coli* O157:H7 (Figure 6; Figure S12, Supporting Information). To the best of our knowledge, there is no comparative metabolomic‐profiling analysis on cationic AMP‐induced stress yet.^[^ ^49^ ^]^ In this regard, a more comprehensive and multi‐dimensional mechanism of molecular responses of bacteria to FCLAPs is available in the present study. We characterized several patterns of bacterial membrane perturbance produced by FCLAPs, including interference with membrane lipid metabolic pathways and key precursors synthesis (Figure 6c). In view of these attractive modes of action, we next sought to define the interactive site between FCLAPs with bacterial cell membranes. Combined with the results of LPS binding assays and structural interaction (Figure 7), we proposed a mechanism for FCLAPs‐LPS interactions that allows the peptides to traverse the LPS layer and target the PGN layer. On this basis, we further performed a panel of phenotypic experiments (Figures 4 and 5), which corroborated our inference indicating that FCLAPs kill bacteria via the dual‐targeting mechanism.

Specifically, we first assert that FCLAPs are initially targeted to LPS, the outermost layer of *E. coli*. We noted that FCLAPs caused gene perturbations in the LPS metabolism pathway, along with a significant drop in the metabolic levels of PPP intermediates, which are crucial precursor metabolites for the biosynthesis of LPS. Intriguingly, our study is the first to report that the FCLAPs caused inhibition of the membrane lipid biosynthetic process by decreasing the key precursor metabolites. This would be a different mechanism from the precedent study which acts via inhibiting the lipid A synthesis or LPS modifications.^[^ ^50^ ^]^ The result agrees well with those seen in LPS binding assays, wherein the exogenous addition of bacteria LPS in the cell membrane promptly diminished the activity of KTA and KTR in a dose‐dependent manner (Figure 7a). The interplay between FCLAPs and LPS was also confirmed by the CD spectra, which recorded the conformational transitions of peptides from disordered into helical states (Figure 7i–k). The results could be explained by a previous study which demonstrated that certain conformation may be a necessary requirement to permeabilize through the LPS layer.^[^ ^51^ ^]^ We also noticed the sequential increase of outer and inner membrane permeability, again signifying that FCLAPs kill *E. coli* by disrupting the outer membrane and inner membrane successively (Figure 5b–e). The results were consistent with a previous mechanism study in which a short linear AMP can trigger membrane damage by binding to the LPS in the outer membrane followed by disrupting the phosphatidylglycerol in the cytoplasmic membrane.^[^ ^3c^ ^]^

Apart from the LPS layer, perturbations on the inner leaflet of the cell membrane were also apparent in FCLAP‐treated cells, wherein expressions of the major genes involved in phospholipids and fatty acids biogenesis were dramatically decreased. These early metabolic signatures of membrane lipid damage agreed with the primary mode of action of FCLAPs, which implicated disorganizing the bacterial membrane through their strong interaction with phospholipids (Figure 7g,h). In addition, we found that following the destabilization of the LPS and phospholipids leaflets, there were concomitant perturbances in the outer membrane, involving loss of membrane integrity, depolarization of membrane potential, and abnormality of ion efflux (Figure 5). These results were in agreement with those reported in the literatures which revealed that some AMPs can disturb the bacterial membrane potential by altering or rearranging the hydrophobic L‐ and D‐amino acid residues and forming ion channels.^[^ ^52^ ^]^ Based upon the data collected, we proposed that following FCLAP‐LPS interaction, the peptides would penetrate the LPS outer leaflet and phospholipid inner leaflet; once traversing the outer membrane, FCLAPs would then reach the PGN layer to begin the destructive process.

Thereafter, the peptidoglycan layer was found to be considerably perturbed as evidenced by the multi‐omics studies. We therefore speculate that the peptides collapsed the membrane homeostasis by targeting the bacterial peptidoglycan. As an essential structure surrounding most bacterial cells, peptidoglycan protects the cells from bursting and provides them with their characteristic shapes.^[^ ^53^ ^]^ However, whether AMP‐peptidoglycan interactions may be one of the determinants of the antibacterial mechanisms is not yet well understood.^[^ ^35a^ ^]^ If this was the case, we attempted to examine if the FCLAPs elicit antibacterial activity through disruption of PGN integrity or synthesis. KTA and KTR‐treated cells demonstrated more marked phenotypes than untreated ones, as illustrated by the increased susceptibilities to lysozyme in the PGN integrity assay and large increments in the cell length in HADA labeling observation (Figure 8a–c). The loss in PGN integrity was consistent with the diminishment in the polysaccharide region spotted in FTIR spectral profiles (Figure 5h). Given that PGN is fundamental for survival, its chemical structure might follow the dynamics developed by the Red Queen hypothesis effect, an evolutionary arms race in which microbes would change the PGN structure in response to specific threats to the cell envelope.^[^ ^54^ ^]^ Herein, our data suggest that KTA and KTR dramatically perturbed the levels of key precursors, especially UDP‐MurNAc‐pentapeptide, in PGN synthesis at a concentration below the MIC (Figure 8d,e). This inhibitory effect on PGN metabolism might explain the filamentous appearance observed in HADA labeling, where the septal cell wall is blocked causing cell elongation without division. We therefore verified PGN metabolism as another potential target of FCLAPs, which to our knowledge is distinct from that of any previously reported cationic AMP.

Last, we need to emphasize the limitations of this work. The interaction modes between FCLAPs and bacterial membrane components should be addressed in more detail. It is also worth mentioning that FCLAPs have an excellent inhibitory effect against two strains of Gram‐positive *S. aureus*. In this regard, their mechanisms of action against Gram‐positive bacteria should be intensively explored, given the strikingly diverse membrane structures between different Gram‐status. Additionally, KTR proves potent against *Klebsiella pneumoniae*, *Acinetobacter baumannii*, and *Pseudomonas aeruginosa*, all of which are recognized as “superbugs” involved in antibiotic‐resistant nosocomial infections. As for the in vivo experiments, it is essential to evaluate if the FCLAPs would cause nonspecific killing of commensal bacteria when applied for therapy. According to the literature, human gut commensals can evolve high resistance to cationic AMPs by neutralizing the negative charge of the cell, decreasing AMP binding at the bacterial surface, and reducing AMP‐dependent membrane disruption. And a growing number of reports demonstrated that AMPs specifically targeting pathogens but not commensal species at therapeutic doses, and the selectivity may provide an important therapeutic advantage over broad‐spectrum antibiotics. Nevertheless, to allow for a more solid conclusion to be made, more investigations are required to shed light on the selective action of the FCLAPs and confirm their effects against the commensal bacteria. Moreover, we have cleared normal flora in the intestinal tracts in prior to the *E. coli* colonization. The method was commonly adopted to test the in vivo activity of antibacterial agents to eliminate any possible interference from microbiota in the intestines. However, the possible influence of natural microbiota on the antimicrobial efficacy of FCLAPs may be positive or negative, and is quite worth of further study. Corresponding research will be carried out at normal physiological environment in our future experiments and reported separately. To this end, we should continue our research to determine the mechanism underpinning its good antibacterial activity and test its potential for clinical translation. Encouraged by the exceptional efficacy in food surface decontamination, FCLAPs also hold promise for expanding application in more food processing scenarios, such as the development of food antimicrobial coatings and packaging. Further medicinal chemistry research would also be needed to increase the potency of FCLAPs and their possibility in food processing and clinical practice.

In summary, the comprehensive modifications of natural AMPs show great potential in obtaining FCLAPs with robust antibacterial performance against important Gram‐negative foodborne pathogens, especially *E. coli* O157:H7. The present study also provides a fine example of how structural modification of a natural AMP derived from a food source impacts its antibacterial activity and cytotoxicity. The superior in vitro antibacterial performance of FCLAPs, in addition to the highly favorable insusceptibility to antibacterial resistance, and the aforementioned distinctive dual‐targeting modes of action, implies a great potential for these peptides in developing antimicrobial agents. More inspiringly, the excellent preclinical safety profiles, as described here, also strongly support further advancement toward therapeutic and food applications.

## 4 Experimental Section

### Bacterial Strains

Ten bacterial strains used in this study were obtained from Guangdong Microbial Culture Collection Center. *E. coli* MDR was a generous gift from Zhejiang Academy of Agricultural Sciences. The *E. coli* O157:H7 (ATCC 35 150), *E. coli* (ATCC 8739), *E. coli* MDR, *Salmonell*a *Typhimurium* (ATCC 14 028), *Klebsiella pneumoniae* (ATCC 13 883), *Acinetobacter baumannii* (ATCC 17 978), *Pseudomonas aeruginosa* (ATCC 10 145), and *Enterobacter cloacae* (ATCC 13 047) were used as representative gram‐negative bacteria. The *Enterococcus faecium* (ATCC 19 434), *Staphylococcus aureus* (ATCC 25 923), and *Staphylococcus aureus* MRSA (ATCC 43 300) were used as representative gram‐positive bacteria. All strains were cultured in nutrient broth (NB) medium at 37 °C with shaking at 180 rpm before use.

### Synthesis of Antimicrobial Peptides

FCLAPs were synthesized according to the standard Fmoc solid‐phase peptide synthesis protocols. The fidelity of the peptides was determined by a triple‐quadrupole time‐of‐flight mass spectrometry (Q‐TOF MS; AB SCIEX, Framingham, MA, USA). The purity was confirmed to be more than 95% by an Agilent 1200 high‐performance liquid chromatography system (HPLC; Agilent technologies, Santa Clara, CA, USA) with an analytical reverse‐phase Agilent ZORBAX SB‐Aq column (4.6 × 250 mm, 5 µm). The peptide detected at 220 nm was eluted with a gradient of acetonitrile (containing 0.1% trifluoroacetic acid) from 5% to 95% at a flow rate of 1 mL min^−1^. Peptide theoretical MW, net charge, and hydrophobic ratio were analyzed using the online tool http://heliquest.ipmc.cnrs.fr/.

### CD Spectroscopy

The CD spectra were recorded on a Jasco J‐1500 spectropolarimeter (JASCO Corporation) using a quartz cylindrical cell (pathlength 1.0 mm). FCLAPs were dissolved in 10 mmol L^−1^ phosphate‐buffered saline (PBS; pH 7.4), 50% (v/v) trifluoroethanol (TFE), or 30 mmol L^−1^ SDS solutions to a final concentration of 0.1 mmol L^−1^. Scans were performed at wavelengths ranging from 190 to 260 nm with a speed of 100 nm per min at 25 °C. Three consecutive scans were averaged, and the spectra of solvents were subtracted from each corresponding peptide spectrum.

CD measurements were also conducted to determine the interaction between FCLAPs (0.1 mmol L^−1^) and LPS (0.5 mg mL^−1^, ≈25 µmol L^−1^). To evaluate the interaction between FCLAPs (0.1 mmol L^−1^) and PGN, the peptides were scanned in the presence of 1 mmol L^−1^ PGN. Spectra were corrected for background scattering by subtracting the spectrum of buffer in the absence of either FCLAPs, LPS, or PGN.

### MIC Measurements

MICs of FCLAPs and antibiotics (tobramycin and colistin) were determined using the broth micro‐dilution method. Briefly, bacterial strains were overnight cultured to stationary phase at 37 °C with aeration at 180 rpm and diluted in Mueller–Hinton broth (MHB) to a cell density of approximately 1.0 × 10^6^ colony forming units (CFU) per milliliter (CFU mL^−1^) as the working suspension. Next, 100 µL aliquots of the suspension were transferred into a UV‐sterilized 96‐well microtiter plate (Corning) containing 100 µL of peptide solutions diluted serially twofold. After incubating the plates at 37 °C for 24 h, the MIC was determined as the lowest concentration at which no bacterial growth could be detected by the eye. All MIC assays were independently repeated in triplicate.

### MBC Assays

The bacterial suspension with a concentration of AMPs superior or equal to the MIC were diluted and transferred to a Plate Count agar (PCA) plate. The MBC value was defined as the minimum concentration to result in no visible colony on the plate after 24 h incubation at 37 °C. The tests were performed in triplicate.

### Time‐Kill Curves

Time‐kill studies with FCLAPs were conducted against *E. coli* O157:H7. Overnight cultures of *E. coli* O157:H7 in nutrient broth were obtained from independent colonies grown on eosin‐methylene blue agar. The bacteria suspension was then diluted 1:1000 in nutrient broth. *E. coli* was treated with 4 × MIC FCLAPs and the time at which each FCLAPs was added was defined as 0 h. Colistin (4 × MIC) was used as a positive control, and sterile PBS was used as a negative control. At each time point, 100 µL aliquots were collected and centrifuged, pellets washed in 100 µL sterile PBS and resuspended in 100 µL PBS and tenfold serially diluted suspensions were plated onto Muller–Hinton agar (MHA). After overnight cultivation at 37 °C, colonies were enumerated and the CFU mL^−1^ was calculated. Time‐kill curves were constructed as the time versus the natural logarithm of the CFU mL^−1^.

### Resistance Studies

The method was adapted from a published procedure.^[^ ^55^ ^]^ *E. coli* was repeatedly exposed to subinhibitory concentrations of FCLAPs by serially passaging them up to 30 steps of successive culture in microplates. Briefly, overnight cultures of *E. coli* O157:H7 were diluted in a geometric progression with nutrient broth medium to a concentration of 10^6^ CFU mL^−1^. The bacteria suspension was then inoculated with fresh nutrient broth containing 1/2 level MIC of FCLAPs, followed by incubation at 37 °C under shaking for 24 h. Colistin was used as a positive control. The real‐time MIC values of the FCLAPs and colistin were measured every two cell‐drug incubation cycle. The concentration of antimicrobials throughout the test was adjusted to keep at 1/2 × MIC. Repeated bacterial cultivations in the presence of FCLAPs or colistin were conducted in triplicate.

### Proteolytic Resistance Assays

Proteolytic resistance of the FCLAPs was evaluated by a protease sensitivity assay, CD measurement, and HPLC analysis as previously described.^[^ ^14^, ^56^ ^]^ The protease sensitivity of the peptides was tested in the presence of pepsin and trypsin. Briefly, a solution of peptide/protease molar ratio of 20:1 or 100:1 was made in 10 mmol L^−1^ PBS (pH 2.0 for pepsin and pH 8.0 for trypsin) and was incubated at 37 °C for 1 h. Melittin was used as a positive control. The MIC values were determined as described above.

For the CD measurement, peptides (0.1 mmol L^−1^) were mixed with the protease solutions (peptide/protease molar ratio of 20:1). After incubation at 37 °C for 1 h, the samples were boiled at 100 °C in a water bath for 30 min. Then, the mixtures were centrifuged at 13 000 g for 30 min to precipitate the proteases, and the supernatant was collected. All the samples for CD spectra were dissolved in 50% TFE solution. The peptides alone were used as a control. For the HPLC analysis, the supernatant (10 µL^−1^) was loaded onto an analytical reverse‐phase Agilent ZORBAX SB‐Aq column for detection as described above.

### MTT Assays

NIH/3T3 fibroblast cells and LO2 cells were cultivated using Dulbecco's modified eagle medium (DMEM) at 37 °C with 5% CO_2_. Next, cells were collected, resuspended, and diluted in DMEM. An aliquot of 100 µL of the cell suspension was seeded into a 96‐well plate at a cell density of 5 × 10^3^ cells per well, followed by incubation for 24 h at 37 °C in a 5% CO_2_ atmosphere. After culture medium removal, fresh DMEM containing FCLAPs or colistin at various concentrations (40–640 µg mL^−1^) was added. After 24 h, a 0.5% volume of methyl thiazolyl tetrazolium (MTT) solution was introduced and incubated for 4 h at 37 °C. Once removing the solution, DMSO was added to dissolve the purple solid. Wells containing DMEM or cells only were used as blank and positive control, respectively. The optical density (OD) in each well was measured at 490 nm on a Multiskan GO microplate reader (Thermo Fisher Scientific, USA). The percentage of cell viability of different treatment groups was calculated from

(1) $$\mathrm{Cell}\;\mathrm{viability}\left(\%\right)=\frac{Abs_{\mathrm{Sample}}-Abs_{\mathrm{Blank}}}{Abs_{\mathrm{Control}}-Abs_{\mathrm{Blank}}}\times 100$$

to calculate the cytotoxicity of FCLAPs.

### Alamar Blue Assay

Alamar Blue Cell Viability Assay Reagent provides a simple, rapid, and reliable method for cell proliferation and cytotoxicity detection.^[^ ^57^ ^]^ According to the manufacturer's protocols, LO2 cells with specific peptide concentration were mixed with detection reagent and incubated in active‐control environmental chamber (37 °C, 5% CO2) for 4 h. Colistin was used as a positive control. The cell cytotoxicity was detected by measuring the relative fluorescence unit (RFU) at excitation at 530 nm and emission at 590 nm.

### PicoGreen Staining Assay

DNA damage was detected by PicoGreen dsDNA quantitation kits purchased from Solarbio (Beijing, China) following the manufacturer's suggestions. Briefly, the content of dsDNA was determined by measuring the fluorescence intensity of the extracted DNA mixed with the fluorescent dye PicoGreen.

### Lactate Dehydrogenase (LDH) Activity Assay

LDH activities were measured by colorimetric assay under 450 nm using specific test kit bought from Solarbio (Beijing, China) according to the manufacturer's protocols.

### Hemolysis Activity

The toxicity level of FCLAPs in regard to hemolytic potential was evaluated based on the standard protocol.^[^ ^58^ ^]^ SD rat blood cells were collected and washed with PBS three times, then 0.5 mL of 5% red blood cells (RBCs) were added to 0.5 mL in different concentrations (1, 2, 4, 8, 16, 32, and 64 × MIC) of various AMPs. Meanwhile, 1% Triton X‐100 and PBS were used as the positive and negative control, respectively. After incubation for 1 h at 37 °C, cells were centrifuged at 3000 g for 10 min. The absorbance of released hemoglobin in the supernatants was measured at 570 nm by a microplate reader (Multiskan GO). The relative hemolysis percentage was calculated by the following equation:

(2) $$\mathrm{Hemolysis}\left(\%\right)=\frac{Abs_{\mathrm{Sample}}-Abs_{\mathrm{Blank}}\;}{Abs_{\mathrm{Positive}\;\mathrm{control}}-Abs_{\mathrm{Blank}}}\times 100$$

### Gut Colonized Model

Female ICR mice aged 6–8 weeks and weighing around 20 g were used for this experiment. Mice were purchased from Hangzhou Medical College (Hangzhou, China), with the laboratory animal production license number SYXK (ZJ) 2019‐0002. Mice were randomly housed (five mice per ventilated cage) and adapted to standardized environmental conditions (25 ± 1 °C and 30 ± 10% humidity in a 12 h light‐dark cycle with free access to water and a basal diet) in Hangzhou Medical College to minimize potential confounders. Mice were maintained in strict accordance with the Regulations on Administration of Animals Used as Subjects of Experiments approved by the State Council of the People's Republic of China (State Council Gazette Supplement, Aug 20, 2017). The animal experiment protocols were conducted in compliance with the relevant guidelines and regulations of Hangzhou Medical College, and the experiments were approved by the Institutional Animal Care and Use Committee. The laboratory animal usage license number is SYXK‐2019‐0011, certified by the Science and Technology Department of Zhejiang Province.

ICR mice (*n* = 10 per group) were administered ad libitum with drinking water containing 5 g L^−1^ streptomycin from day 1 to day 5. On day 6, mice went through intragastric administration with *E. coli* O157:H7 (2.0 × 10^8^ CFUs). At 24 h after introduction of the inoculums, mice were orally administered by an intragastric savage with 200 µL PBS, KTA (20 mg kg^−1^ BW, 9.87 µmol L^−1^), KTR (40 mg kg^−1^, 18.95 µmol L^−1^), RI (60 mg kg^−1^ BW, 38.31 µmol L^−1^), RT (40 mg kg^−1^ BW, 24.81 µmol L^−1^), or ciprofloxacin (10 mg kg^−1^ BW, 30.18 µmol L^−1^). A blank group, in which the mice were neither colonized with *E. coli* nor treated, was also included. Six mice from each group were used for the endpoint experiments, three for histological evaluation, and the rest for backup. The treatments were applied for 7 consecutive days. Fecal pellets aseptically collected on days 1, 3, 5, 7, and 9 post‐colonization were directly resuspended in normal saline to count the bacterial burdens. Mice were humanely euthanized at the experimental endpoint of FCLAPs or antibiotic treatments (*n* = 6 mice for each group). Lumenal contents of the ileum, cecum, and colon were aseptically expelled, weighed, homogenized, and enumerated for bacterial loads after plating on MacConkey agar. All results were expressed as the logarithm of CFU per gram of fecal pellets (CFU g^−1^).

### Histological Evaluation

Ileum, cecum, and colon segments from mice (*n* = 3) in different groups were subjected to histological examination. The tissues were fixed in 4% paraformaldehyde solution overnight followed by gradient ethanol dehydration and paraffin‐embedding. Next, the specimens were sectioned (3 µm thick) into slides, which were stained with hematoxylin and eosin (H&E). The slides were observed under an optical microscope and photographed at appropriate magnification. After observation, the histological grading was assessed following the standard scoring criteria as shown in Table S3, Supporting Information.^[^ ^59^ ^]^

### Food Surface Decontamination Model

To determine the potential of FCLAPs as food preservatives, a food surface decontamination model contaminated by *E. coli* O157:H7 was established. Briefly, cherry tomatoes (*n* = 9 per group) were sanitized and dried followed by spot‐inoculated with 50 µL aliquots of *E. coli* O157:H7 suspension (1.0 × 10^8^ CFUs). The skin surface (1.0 cm × 1.0 cm) with the inocula was marked with an indelible pen. After incubation for 2 h (allowing attachment of the *E. coli* on the fruit surfaces), the marked sections were aseptically removed with sterilized scissors. Each piece was immersed in 2 mL sterile PBS, KTA (50 µg mL^−1^), KTR (100 µg mL^−1^), RI (150 µg mL^−1^), or RT (100 µg mL^−1^) solutions for 10 min. Afterward, three pieces under the same treatments (weighing around 0.5 g) were combined, homogenized using a stomacher device, and spread on PCA plates to count the bacterial burdens after incubation at 37 °C for 17 h. The microbiological data were recorded as CFU per gram of cherry tomato skins (CFU g^−1^).

### Morphology Observations

The surface morphological alterations and ultrastructural changes of *E. coli* O157:H7 cells upon FCLAP treatments were observed by a SU‐8010 scanning electron microscope (Hitachi, Japan) and an H‐7650 transmission electron microscopy (Hitachi, Japan), respectively. The sample preparation and observation were performed according to the previous report.^[^ ^34^ ^]^

### Fluorescence Microscopy

The physiological effects of FCLAPs on bacterial cells were evaluated using visualized fluorescent imaging. *E. coli* O157:H7 was cultured in MHIIB until the stationary phase. Cells were then incubated with FCLAPs (4 × MIC) for 1 h. Following AMP treatment, cells were stained with 20 µg mL^−1^ FM4‐64 (Molecular Probes) and 10 µg mL^−1^ DAPI (Biofroxx) for 10 min. Each stained culture was then centrifuged and resuspended in 1/10 volume of the original cultures. Five microliters of the concentrated cells were spotted onto a 1.5% agarose‐coated glass slide for microscopy. Images were collected using a ZEISS LSM 880 super‐resolution confocal microscope using a 63 × oil‐immersion objective lens.

### Detection of Nucleic Acid and Protein Leakage

The leakage of intracellular protein and nucleic acid was determined using a previously described method.^[^ ^60^ ^]^ Treated and non‐treated (control) cell suspensions were centrifuged for the collection of supernatants, which were then transferred into new aseptic Eppendorf tubes prior to the cytoplasm leakage assays.

The content of nucleic acid release was measured by reading the absorbance of the supernatant utilizing a UV–vis spectrophotometer (UV‐2600, Shimadzu, Tokyo, Japan), and the results were presented in terms of OD_260 nm_. Simultaneously, the amount of released proteins was calculated using OD_280 nm_, and a standard curve was generated using bovine serum albumin (BSA) standard solutions.

### Transcriptomics and RT‐qPCR Analysis

Overnight *E. coli* O157:H7 cultures were collected and treated with 4 × MIC of each FCLAP for 1 h. Cell harvest, total RNA extraction, and cDNA library construction were performed following the protocol of the previous study.^[^ ^61^ ^]^ For transcriptome analysis, the purified cDNA library was sequenced using an Illumina Novaseq platform, generating 150 bp paired‐end reads.

The expression of selected DEGs was verified by RT‐qPCR using Power SYBR@Green PCR Master Mix (Applied Biosystems, USA) and a CFX384 Real‐time PCR instrument (Bio‐Rad Laboratories, USA). A comparative critical threshold value (2^−ΔΔCT^) method was used to assess the changes in transcription levels. The primers of selected DEGs in this study were listed in Table S4, Supporting Information.

### Metabolomics Analysis

An untargeted metabolomics analysis was performed to explore the mechanisms of actions of FCLAPs against *E. coli* O157:H7 using a treatment concentration of 4 × MIC. Untreated and treated cells were grounded with liquid nitrogen and resuspended with methanol (80%, v/v). After centrifuging, the supernatant was diluted with LC‐MS grade water to a final concentration of 50%, followed by injecting it into the LC‐MS/MS system analysis. Univariate analysis using a *t*‐test was performed to calculate the statistical significance (*p*‐value). Metabolites with a VIP value > 1.0, FC > 1.2 or < 0.833, and *p*‐value < 0.05 were assigned as potential differential metabolites between the AMP‐treated and control samples.

### Flow Cytometry

Flow cytometry was applied to quantitatively evaluate the membrane disruption activities of the AMPs. Briefly, E. coli ATCC8739 cells cultured to stationary phase were incubated with AMPs at 4 × MIC for 1 or 6 h at 37 °C. The mixtures were centrifuged, and the pellets were washed and resuspended to an optical density at 600 nm (OD_600 nm_) of 0.5. cFDA (Invitrogen Detection Technologies, USA) was added to each sample with the final concentration of 50 µmol L^−1^ and incubated for 15 min at 37 °C. Next, 30 µmol L^−1^ PI (Invitrogen Detection Technologies, USA) in an ice bath was added and incubated for 10 min. After centrifugation, cells were harvested, washed, and resuspended in PBS. Data acquisitions of 2 × 10^4^ bacterial cells were performed with a Gallios flow cytometer (Beckman Coulter Inc., USA) at a laser excitation wavelength of 488 nm. PI‐stained cells emit red fluorescence at 620 nm, whereas green fluorescence was emitted by cFDA at 525 nm.

### Membrane Properties Analysis

OM permeability of *E. coli* O157:H7 upon FCLAPs treatments was determined by the amount of *N*‐Phenyl‐1‐naphthylamine (NPN) uptake. IM permeability was assessed by measuring the release of cytoplasmic *β*‐galactosidase using *o*‐nitrophenol‐*β*‐D‐galactopyranoside (ONPG) as the substrate. *E. coli* O157:H7 cells were incubated with ONPG (30 mmol L^−1^) in the absence or presence of 1 ×, 4 ×, 8 ×, or 16 × MIC FCLAPs at 37 °C. The production of *o*‐nitrophenol (ONP) over 8 h was monitored with a Multiskan GO microplate reader at OD_420 nm_. Membrane potential was detected by the fluorescence analysis of Rhodamine 123. These assays were performed as previously described.^[^ ^34^ ^]^

### Detection of K^+^ Out‐Diffusion

The K^+^ out‐diffusion test was performed based on the previously reported method.^[^ ^62^ ^]^ The collected *E. coli* cells were washed and resuspended in a sterile saline solution. The suspensions were then treated with different disinfection approaches. After that, the mixture was filtered through 0.22 µm PVDF membranes to remove bacterial cells. The concentration of potassium ions in the supernatant was determined using an AA240 flame atomic absorption spectrophotometer (Agilent Technologies, Santa Clara, CA, USA).

### FTIR Spectroscopy

FTIR analyses were conducted using frozen untreated and FCLAPs‐treated samples, and performed by a spectrophotometer (Nicolet iN10 FT‐IR, Thermo Fisher Scientific, Waltham, MA, USA) with the absorption spectra recorded within the range of 4000–400 cm^−1^. Background obtained from the pure potassium bromide (KBr) disc was automatically subtracted.

### LPS, Cationic Ion, and Peptidoglycan Binding Assays

The LPS, cationic ion, and peptidoglycan binding assays were performed based on the MIC assay. Aliquots (100 µL) of *E. coli* O157:H7 suspensions (1 × 10^6^ CFU mL^−1^) were transferred into a 96‐well plate containing purified LPS from *E. coli* O55:B5 (0.5–1000 µg mL^−1^) and FCLAPs. The concentrations of the FCLAPs that inhibited *E. coli* O157:H7 growth were determined in the absence or presence of LPS.

### Phospholipids Binding Assays

The phospholipids binding assays were performed based on the MIC assay. Aliquots (100 µL) *E. coli* O157:H7 suspensions (1 × 10^6^ CFU mL^−1^) were transferred into a 96‐well plate containing purified PE and PG (1–100 µg mL^−1^) and FCLAPs. The concentrations of the FCLAPs that inhibited *E. coli* O157:H7 growth were determined in the absence or presence of phospholipids.

### PGN Integrity Assays

The peptidoglycan integrity was assessed by measuring susceptibility to lysozyme‐mediated bacterial lysis.^[^ ^63^ ^]^ After incubation overnight, log‐phase cultures were prepared in fresh nutrient broth in the absence or presence of KTA (0.625 µmol L^−1^, 1/4 × MIC) or KTR (1.18 µmol L^−1^, 1/4 × MIC) and then incubated for 2 h. Cells were resuspended in 10 mm HEPES (pH 7.5) and adjusted to an OD_600 nm_ of 2.0. Untreated and treated cell suspensions were mixed with an equal volume of HEPES buffer and 10 mm EDTA. After the addition of 1 mg mL^−1^ lysozyme, cell lysis was assessed over 1 h at 25 °C by monitoring OD_600 nm_ every 2 min using a Multiskan GO microplate reader.

### HADA Labeling

A previously described protocol for tracking the progression of peptidoglycan biosynthesis by HADA labeling was adapted for the present study.^[^ ^42a^ ^]^ Overnight cultured bacteria suspensions were added to fresh nutrient broth containing KTA (0.625 µmol L^−1^, 1/4 × MIC) or KTR (1.18 µmol L^−1^, 1/4 × MIC) and grown for 2 h at 37 °C to obtain a log‐phase culture. Fluorescence‐labeled D‐amino acids (HADA) were added to a final concentration of 0.25 mmol L^−1^ and incubated at 37 °C while shaking for 30 min. Cells were then washed and eventually resuspended in PBS. The cells were fixed by a 3% formaldehyde solution and immediately imaged using the confocal laser scanning microscope. Images were acquired with excitation at 395 nm and emission at 435 nm. Samples were taken in 4 biological replicates.

### PGN Intermediates Analysis

The intermediates in PGN synthesis were measured in KTA and KTR‐treated *E. coli* as previously described with minor modification.^[^ ^64^ ^]^ In brief, log‐phase *E. coli* O157:H7 was split into 20‐mL aliquots and exposed to sub‐MIC test antibacterial agents (0.625 µmol L^−1^ KTA, 1.18 µmol L^−1^ KTR, or 2.86 µmol L^−1^ ampicillin as positive control). After 1 h incubation at 37 °C with shaking, cells were pelleted and normalized the OD_600 nm_ to 2.0. The bacterial pellets were washed and centrifuged to remove residual extracellular metabolites and medium components. For extraction, cell pellets were added with a cold extraction solvent (chloroform‐methanol‐water at 1:3:1, vol/vol), frozen in liquid nitrogen, thawed on ice, then vortexed to release the intracellular metabolites. Finally, the samples were centrifuged, and 200 µL of the supernatant was taken out. The analysis was performed with a Waters ACQUITY ultrahigh‐performance liquid chromatography (UHPLC) system coupled to a Triple TOF 5600 mass spectrometer analysis (AB SCIEX, USA). The results reported were representative of four independent experiments.

### Statistical Analysis

All data reported were expressed as mean ± standard deviation (SD). Statistical analysis was conducted using SPSS software (SPSS 25.0 for MAC; IBM Co., USA). Comparisons between groups were analyzed through one‐way analysis of variance (ANOVA) or unpaired Students’ *t*‐test, where levels of differences were defined at *p* < 0.05. A minimum of three independent experiments (biological replicates) of the antibacterial assay were conducted, and three technical replicates were used in each experiment for each bacterium, antibacterial agent, and concentration.

## Conflict of Interest

The authors declare no conflict of interest.

## Supporting information

Supporting Information

Supplementary Table 1

## Data Availability

The data that support the findings of this study are available from the corresponding author upon reasonable request.;
