# Supplementary material for: Combating Escherichia coli O157:H7 with Functionalized Chickpea‐Derived Antimicrobial Peptides
Source: Adv Sci (Weinh). 2022 Dec 23;10(6):2205301. doi: 10.1002/advs.202205301 (PMC9951321; doi:10.1002/advs.202205301)
Supplement: Supplementary file 1 — Supporting Information [file ADVS-10-2205301-s001.pdf]

Supporting Information

Table S1. Minimal bactericidal concentration of functionalized chickpea-derived Leg2 antimicrobial peptides (FCLAPs).

Table S2. Minimal bactericidal concentration (μmol L^{-1}) of FCLAPs against *E. coli* O157:H7 in the presence of digestive proteases.

Table S3. Evaluation criterions of histological injury.

Table S4. Primer sequences for RT-qPCR.

Table S5. Differentially expressed genes and modulated metabolites detected by the integrated transcriptomic and metabolomics analysis.

Figure S1. HPLC and MS data for FCLAPs. Related to Figure 1.

Figure S2. CD spectra of RI and RT and the secondary structure analyses of FCLAPs. Related to Figure 1.

Figure S3. CD spectra of RI and RT treated with proteases. Related to Figure 1.

Figure S4. HPLC spectra of the FCLAPs in the absence or presence of proteases. Melittin was used as a control. Related to Figure 1.

Figure S5. Cytotoxicity evaluation of the parental peptide and FCLAPs on LO2 and NIH/3T3 cell lines by MTT assays. Related to Figure 2.

Figure S6. Hemolytic activity of FCLAPs. Related to Figure 2.

Figure S7. Effects on histopathologic analysis of *E. coli* O157:H7-colonized mouse intestinal compartments. Related to Figure 3.

Figure S8. Representative SEM and TEM images of *E. coli* O157:H7 before and after treatments with RI and RT. Related to Figure 4.

Figure S9. Representative CLSM images of *E. coli* O157:H7 before and after treatments with FCLAPs. Related to Figure 4.

Figure S10. RT-qPCR analysis of the relative expression levels of representative genes in *E. coli* cells treated with KTA (a), KTR (b) for different time.

Figure S11. RT-qPCR analysis of the relative expression levels of representative genes in *E. coli* cells treated with KTA (a), KTR (b), RI (c), or RT (d) compared with the control.

Figure S12. Metabolic perturbation of *E. coli* O157:H7 treated with KTR, RI, and RT. Related to Figure 6.

Figure S13. Flow cytometry dot plot of untreated *E. coli* O157:H7 which were stained by cFDA (*x*-axis) and PI (*y*-axis). Related to Figure 5.

Figure S14. KTR, RI, and RT permeabilize both outer and inner membrane of *E. coli*. Related to Figure 5.

Figure S15. FTIR spectra (4000-400 cm^{-1} wavenumber) and the second derivative of the absorbance (1675-1600 cm^{-1}, 1575-1525 cm^{-1}, and 1475-1375 cm^{-1} wavenumber) of cells treated with KTA (a), KTR (b), RI (c), and RT (d). Related to Figure 5.

Figure S16. RI and RT exert antibacterial effects through peptide-membrane interaction. Related to Figure 7.

Figure S17. LCMS and LCMS/MS analyses of peptidoglycan precursor UDP-MurNAc-pentapeptide. Related to Figure 8.

Table S1. Minimal bactericidal concentration of functionalized chickpea-derived Leg2 antimicrobial peptides (FCLAPs).

Organisms	MBC [μmol L^{-1}]			
	KTA	KTR	RI	RT
Escherichia coli O157:H7 ATCC 35150	9.9	7.1	12.8	15.5
Escherichia coli ATCC 8739	24.7	23.7	63.9	62.0
Escherichia coli MDR[a]	14.8	9.5	22.4	18.6
Salmonella Typhimurium ATCC 14028	49.4	23.7	127.7	93.1
Klebsiella pneumoniae ATCC 13883	49.4	11.8	127.7	31.0
Acinetobacter baumannii ATCC 17978	7.4	4.7	25.5	15.5
Pseudomonas aeruginosa ATCC 10145	98.7	23.7	95.8	124.1
Enterobacter cloacae ATCC 13047	148.1	165.8	255.4	217.1
Enterococcus faecium ATCC 19434	74.1	94.8	191.6	93.1
Staphylococcus aureus ATCC 25923	49.4	47.4	159.6	62.0
Staphylococcus aureus MRSA ATCC 43300	12.3	11.8	127.7	62.0

Note: [a] multi-drug resistant clinical isolate.

KTA, KTR, RI, and RT stands for RI<u>KTA</u>TWRLALRWLKL, RI<u>KTR</u>TWRLALRWLKL, <u>RI</u>KRLALRWLKL, and <u>RT</u>RTWRLALRWLKL, respectively.

Table S2. Minimal bactericidal concentration (μmol L^{-1}) of FCLAPs against *E. coli* O157:H7 in the presence of digestive proteases.

Peptides	Control[a]	Peptide/pepsin molar ratio		Peptide/trypsin molar ratio	
		20:1	100:1	20:1	100:1
KTA	2.5	4.9	2.5	> 49.4	2.5
KTR	4.7	4.7	4.7	> 47.4	4.7
RI	9.6	12.8	9.6	> 63.9	9.6
RT	6.2	12.4	6.2	> 62.0	6.2
Melittin	12.5	> 100.0	> 100.0	> 100.0	> 100.0

Note: [a] The control MIC values were determined in the absence of proteases.

Table S3. Evaluation criterions of histological injury.

Score	Severity of inflammation	Extent of inflammation	Crypt damage
0	None	None	None
1	Mild	Mucosal	Basal 1/3
2	Moderate	Mucosal and submucosal	Basal 2/3
3	Severe	Transmural	Crypts lost but surface epithelium present
4	Severe	Transmural	Crypts and surface epithelium lost

Table S4. Primer sequences for RT-qPCR.

Gene	Primer Sequences (5' to 3')	Size [bp]
*cls*B	F: GGGTTAAAGCGGAAGTCTTGCT<br>R: CTGCCGTCAGTTCATTGACAAAC	78
*fad*E	F: CATCACCCTGCCTTCCAACT<br>R: CCCTTCCATCTTACCGATAGAG	112
*pag*P	F: GCTAACGCAGATGAGTGGATGA<br>R: GCCAGGTGATGGCAGGAAT	100
*ept*A	F: GCCAGCTGGTCATGGTACT<br>R: GCCGACAATCAGGATGGTCAA	129
*mur*A	F: CGCGACCATCAAACTGGAAGA<br>R: GGTTGCAGCACACATGATGGT	124
*mrc*B	F: GAAGCTGCTGGAGGCAACA<br>R: GGCGAATCATCTCAATGCTGTTG	95
*gal*U	F: CCGGTAGTGGGTGATGAAC<br>R: CTGCCAGGTTATCCTGTGACA	91
*gap*A	F: GGTCTGTTCCTGACTGACGAA<br>R: CGATGTCCTGGCCAGCATAT	142
E. coli 16S rRNA	F: CTTCGGGAACCGTGAGACAG<br>R: GCGCTCGTTGCGGGACTTAA	82

Note: F, forward primer; R, reverse primer; bp, base pairs.

Table S5. Differentially expressed genes and modulated metabolites detected by the integrated transcriptomic and metabolomics analysis.

Refer the attached Excel spreadsheet.

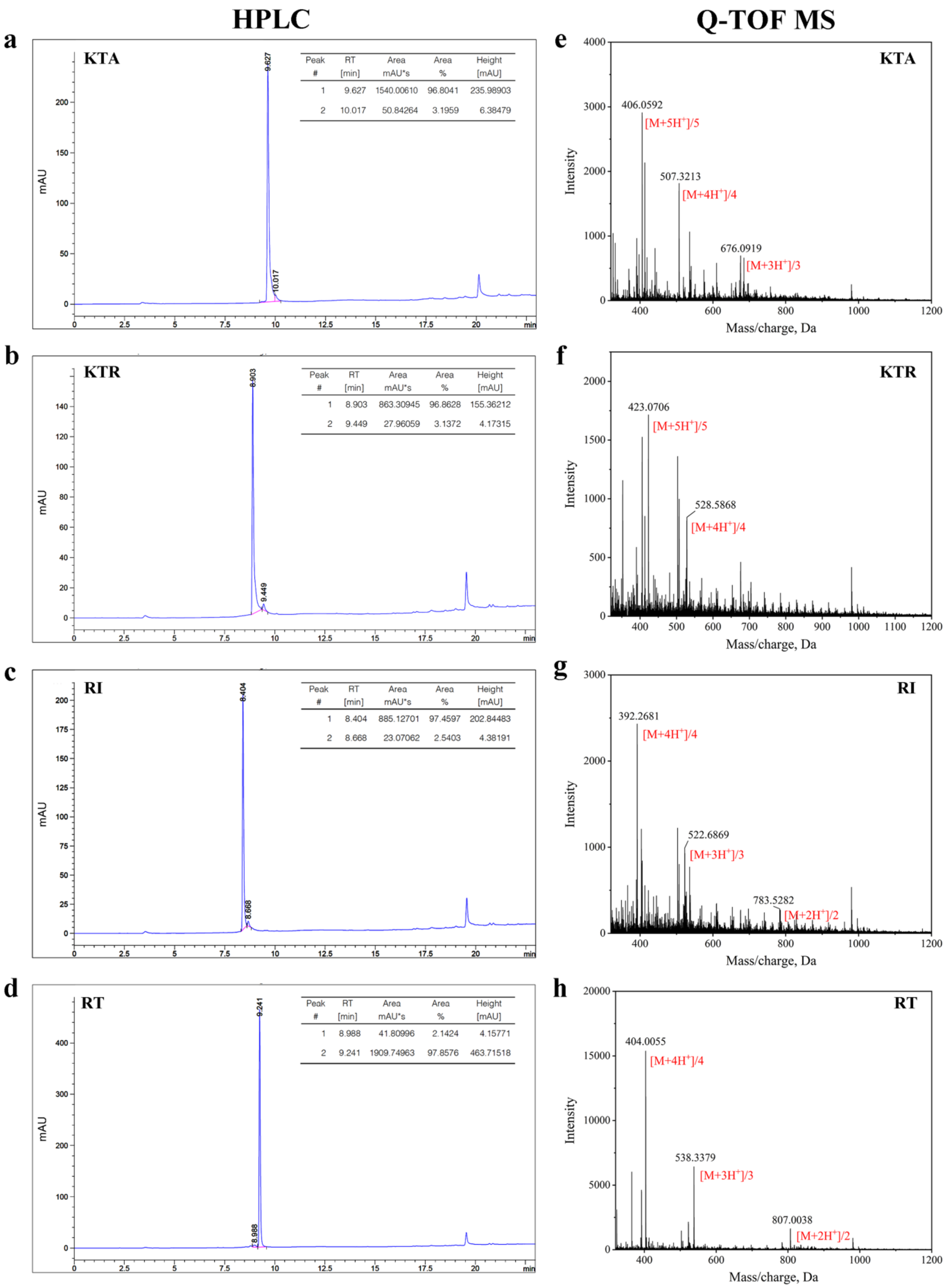

Figure S1. HPLC and MS data for FCLAPs. Related to Figure 1.

a-d) HPLC spectra of KTA (a), KTR (b), RI (c), and RT (d). **e-h)** Q-TOF MS of KTA (a), KTR (b), RI (c), and RT (d).

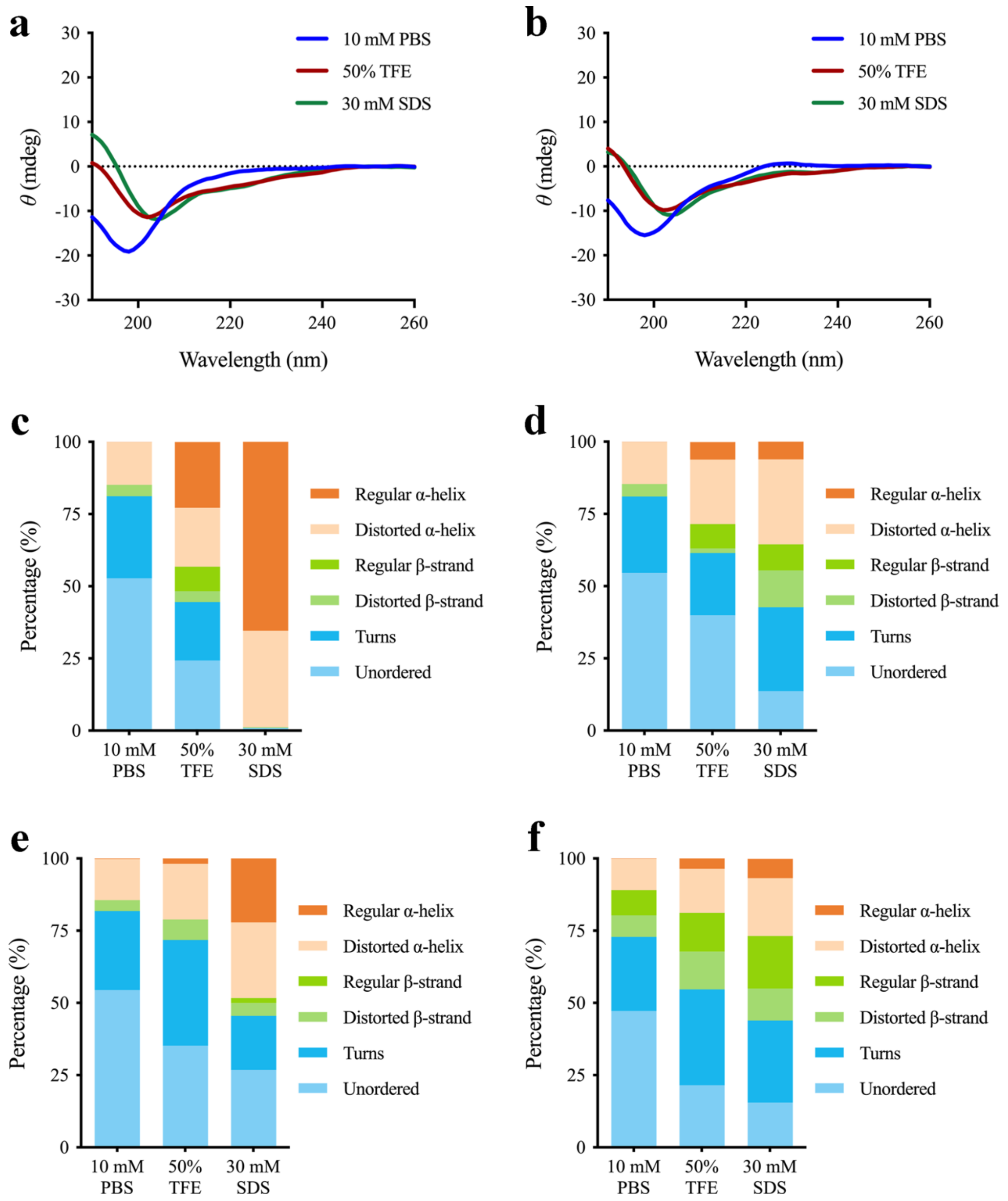

Figure S2. CD spectra of RI and RT and secondary structure analyses of FCLAPs. Related to Figure 1.

a-b) Circular dichroism (CD) spectra of RI and RT, respectively. The peptides were dissolved in 10 mmol L^{-1} phosphate-buffered saline (PBS; pH 7.4), 50% (v/v) trifluoroethanol (TFE), or 30 mmol L^{-1} sodium dodecyl sulfate (SDS). The peptide concentrations were fixed at 0.1 mmol L^{-1}. **c-f)** Abundance ratio of secondary structure motifs analyzed from CD spectral data of KTA (c), KTR (d), RI (e), and RT (f).

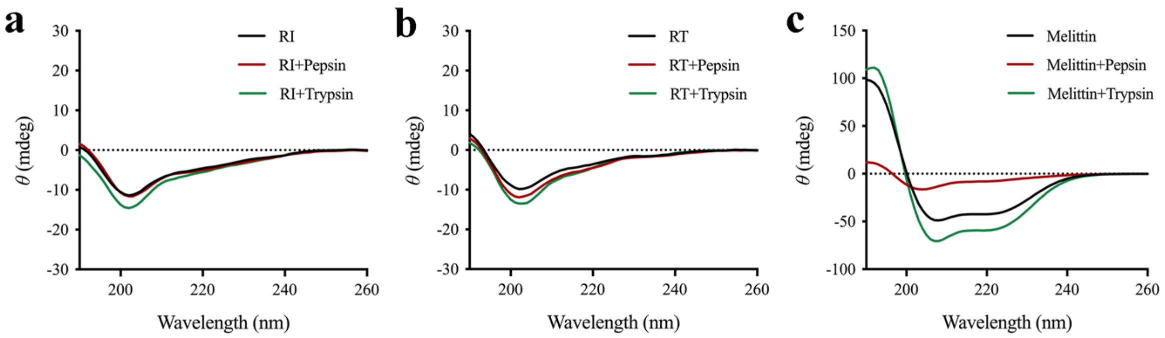

Figure S3. CD spectra of RI and RT treated with proteases. Related to Figure 1.

The peptide concentrations were fixed at 0.1 mmol L^{-1}. All of the samples were dissolved in a 50% TFE solution. The peptide/protease molar ratio was 20:1. Melittin was used as a positive control.

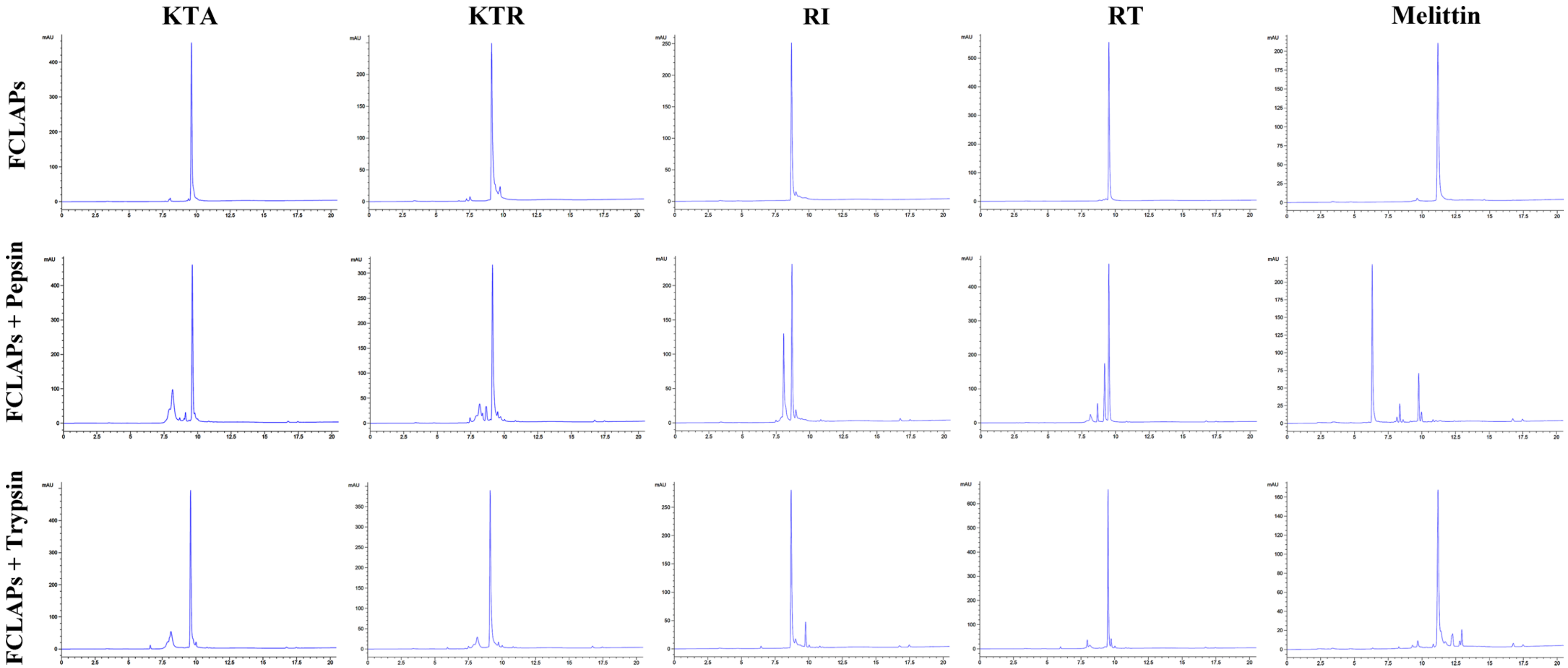

Figure S4. HPLC spectra of the FCLAPs in the absence or presence of proteases. Related to Figure 1.

The peptide concentrations were fixed at 0.1 mmol L^{-1}. The peptide/protease molar ratio was 20:1. Melittin was used as a positive control.

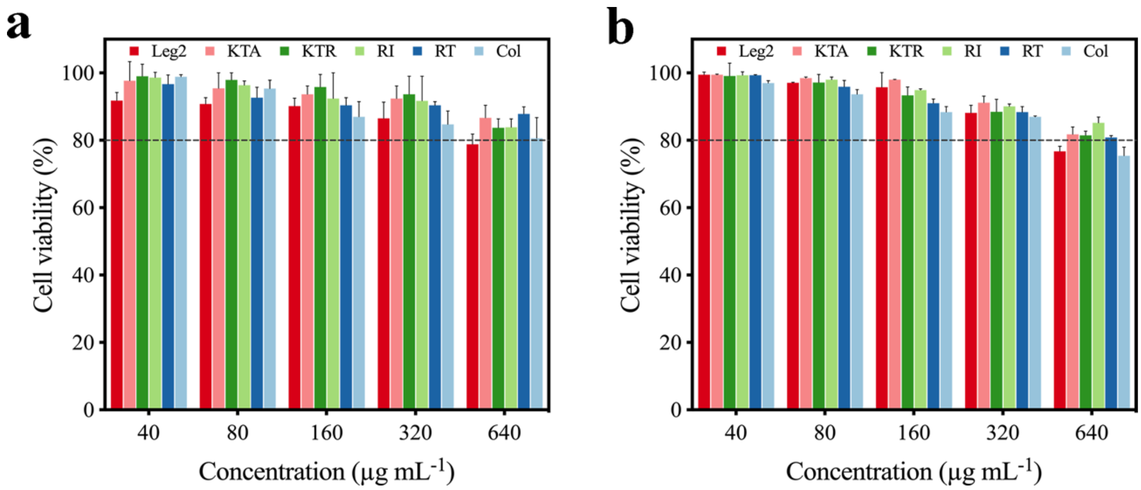

Figure S5. Cytotoxicity evaluation of the parental peptide and FCLAPs on LO2 and NIH/3T3 cell lines by MTT assays. Related to Figure 2.

Cytotoxicity of FCLAPs and parental peptide Leg2 was evaluated using MTT assay on the human LO2 (a) and NIH/3T3 (b) cell lines, respectively. Colistin was used as a control. The tested mass concentrations of the peptides and colistin were 40 − 640 μg mL^{-1}. The corresponding molar concentrations of Leg2, KTA, KTR, RI, RT, and colistin were 18.5 − 296.6 μg mL^{-1}, 19.0 − 303.2 μg mL^{-1}, 19.7 − 316.0 μg mL^{-1}, 25.5 − 408.7 μg mL^{-1}, 24.8 − 397.0 μg mL^{-1}, and 14.8 − 236.6 μg mL^{-1}, respectively. Data are presented as mean ± standard deviation (*n* = 3).

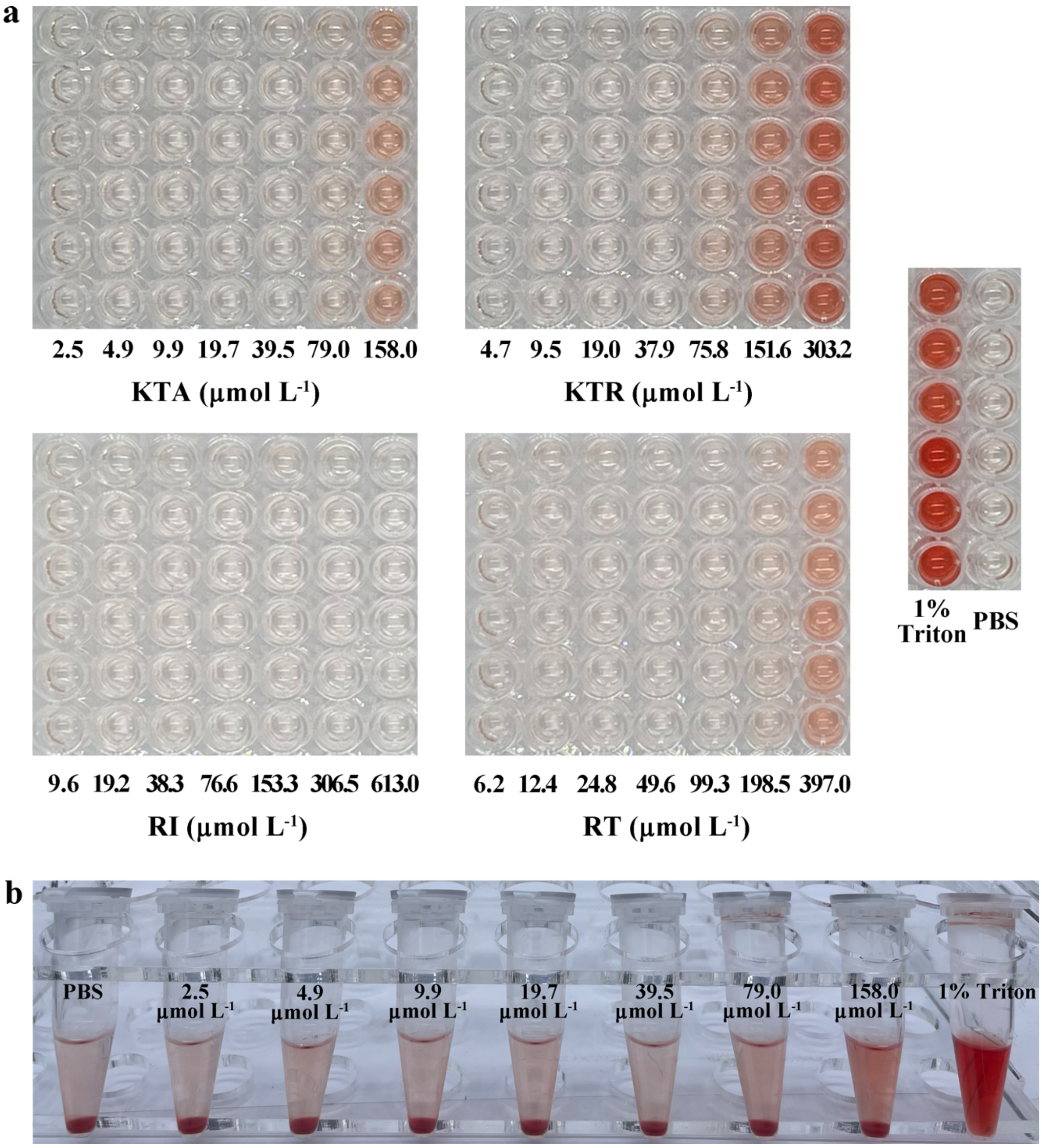

Figure S6. Hemolytic activity of FCLAPs. Related to Figure 1.

a) Hemolytic activity of FCLAPs at different concentrations (1 ×, 2 ×, 4 ×, 8 ×, 16 ×, 32 ×, and 64 × MIC) to the red blood cells of rat. **b)** Hemolytic activity of the KTA at different concentrations (0-158.0 μmol L^{-1}) to the red blood cells of rat. PBS and 1% Triton were used as negative and positive control, respectively. All images are representative of three independent experiments.

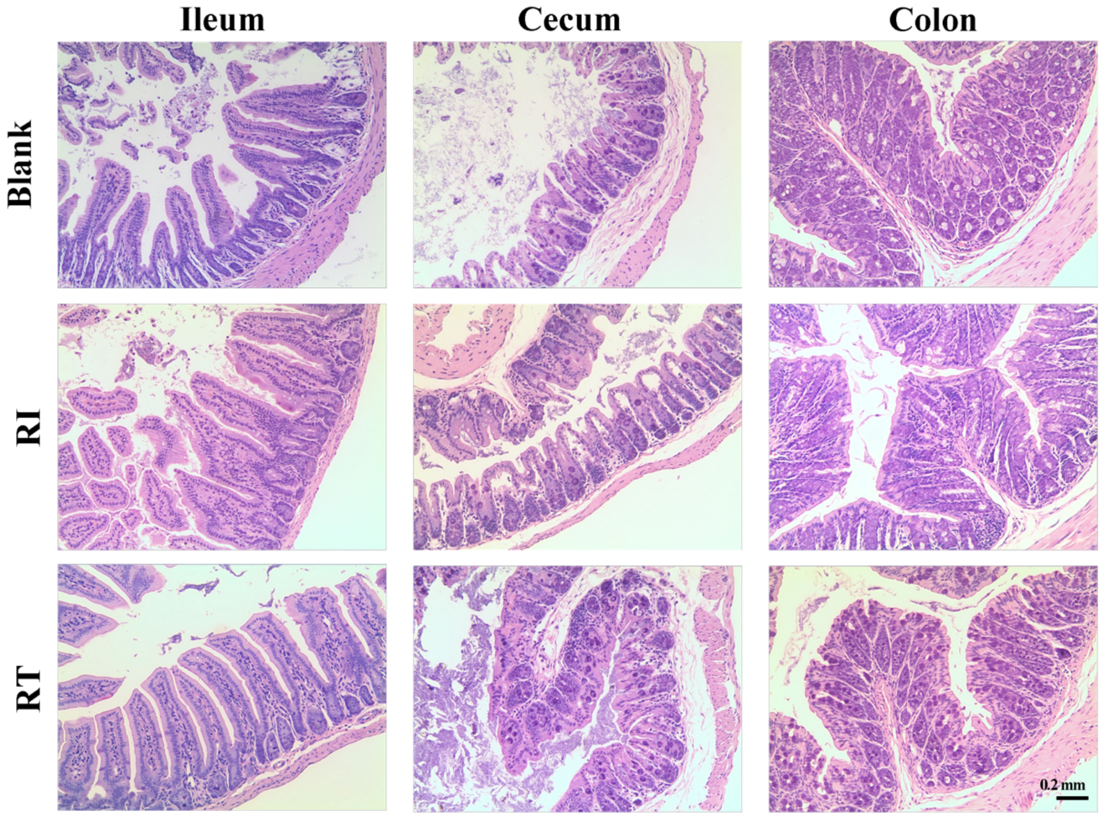

Figure S7. Effects on histopathologic analysis of *E. coli* O157:H7-colonized mouse intestinal compartments. Related to Figure 3.

H&E staining images (magnification × 100) of ileums, cecums, and colons in different groups after treatment for 7 days. A scale bar represents 200 µm. All images are representative of three independent observations.

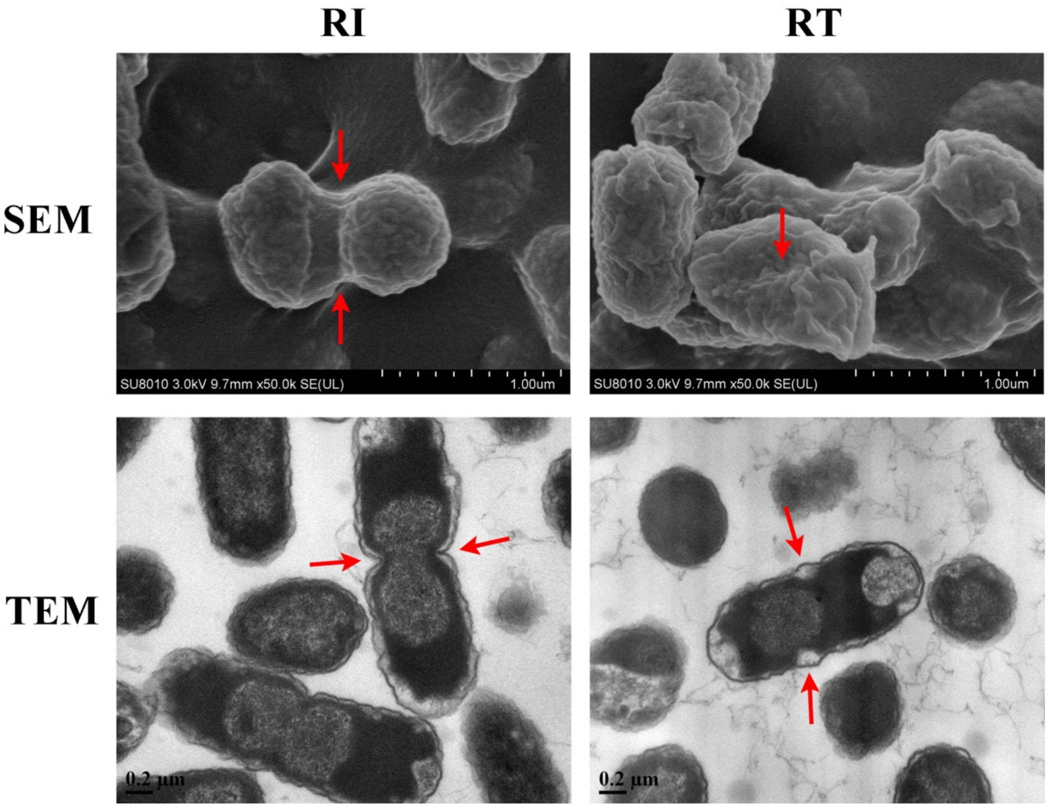

Figure S8. Representative SEM and TEM images of *E. coli* O157:H7 before and after treatments with RI and RT. Related to Figure 4.

E. coli O157:H7 cells were treated with 4 × MIC RI and RT for 1 h before observation, respectively. For SEM images, a scale bar represents 1.0 μm. For TEM images, a scale bar represents 0.2 μm. Regions of interest are indicated by red arrows. All images are representative of three biologically independent experiments performed with similar results.

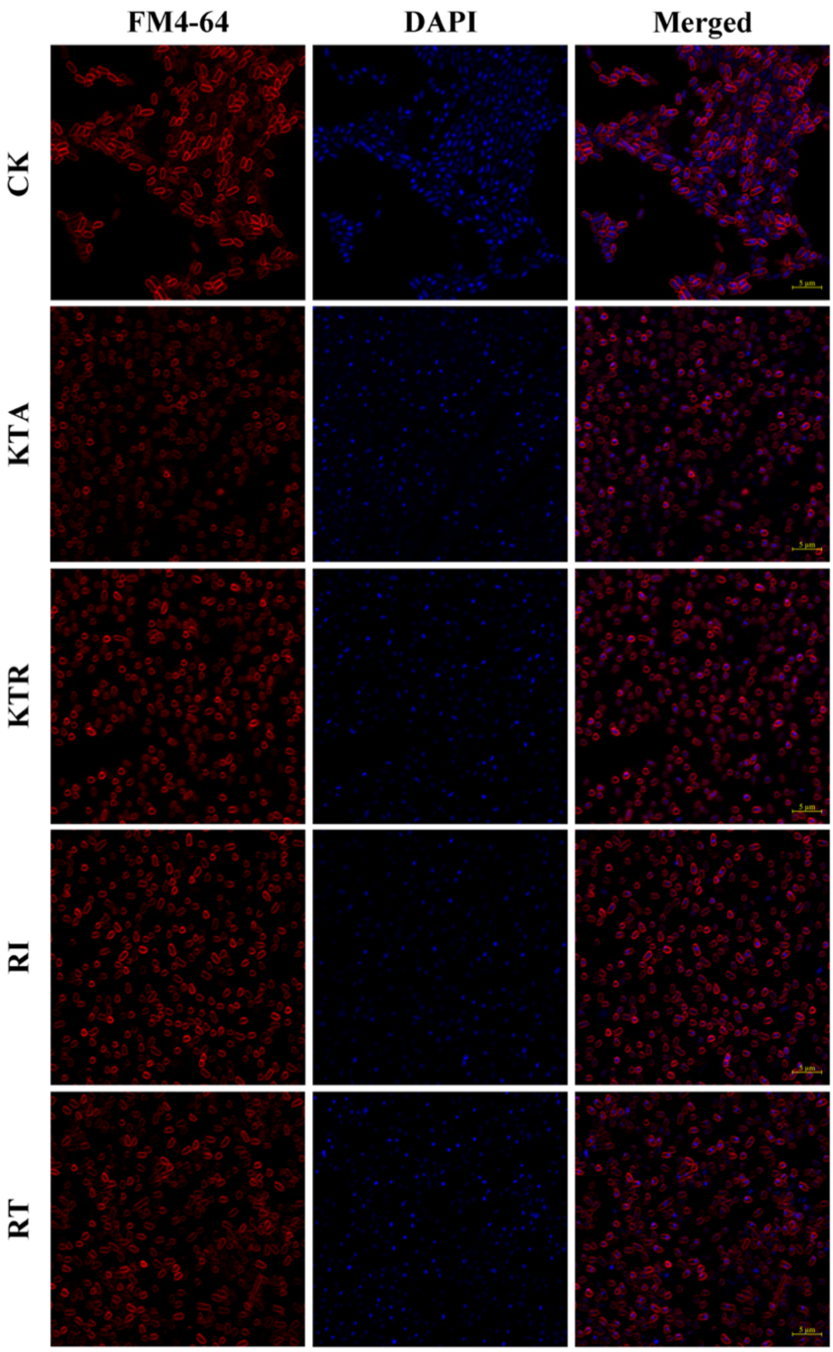

Figure S9. Representative CLSM images of *E. coli* O157:H7 before and after treatments with FCLAPs. Related to Figure 4.

Images of *E. coli* O157:H7 incubated with FCLAPs at 4 × MIC for 1 h. The *E. coli* cell membrane and nucleoid were stained with FM4-64 (red) and DAPI (blue), respectively, in all images. A scale bar in the CLSM image represents 5.0 μm. All images are representative of three independent experiments.

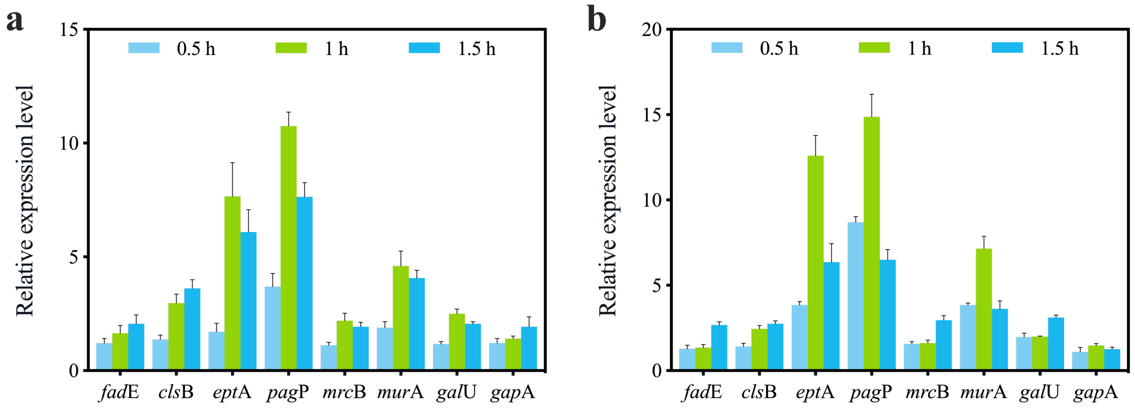

Figure S10. RT-qPCR analysis of the relative expression levels of representative genes in *E. coli* cells treated with KTA (a), KTR (b) for different time.

E. coli cells were treated with 4 × MIC concentrations of KTA (a) or KTR (b) for 0.5 h, 1 h, and 1.5 h, respectively. *fad*E is a representative gene in fatty acid metabolism. *cls*B is a representative gene in phospholipid metabolism. *ept*A and *pag*P are representative genes in lipopolysaccharide biosynthesis. *mrc*B and *mur*A are representative genes in peptidoglycan biosynthesis. *gal*U is a representative gene in sugar metabolism. *gap*A is a representative gene in energy metabolism. Data are presented as mean ± standard deviation (*n* = 3).

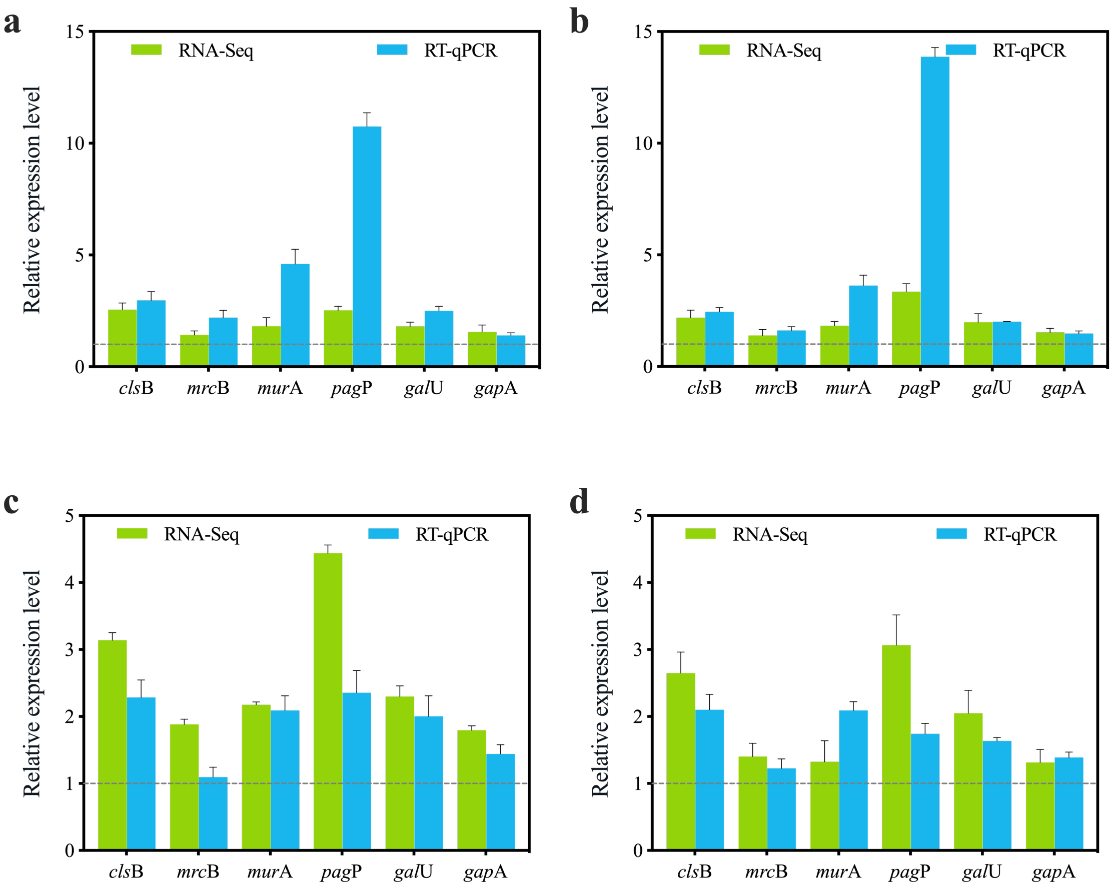

Figure S11. RT-qPCR analysis of the relative expression levels of representative genes in *E. coli* cells treated with KTA (a), KTR (b), RI (c), or RT (d) compared with the control. *cls*B is a representative gene in phospholipid metabolism. *mrc*B and *mur*A are representative genes in peptidoglycan biosynthesis. *pag*P is a representative gene in lipopolysaccharide biosynthesis. *gal*U is a representative gene in sugar metabolism. *gap*A is a representative gene in energy metabolism. Data are presented as mean ± standard deviation (n = 3).

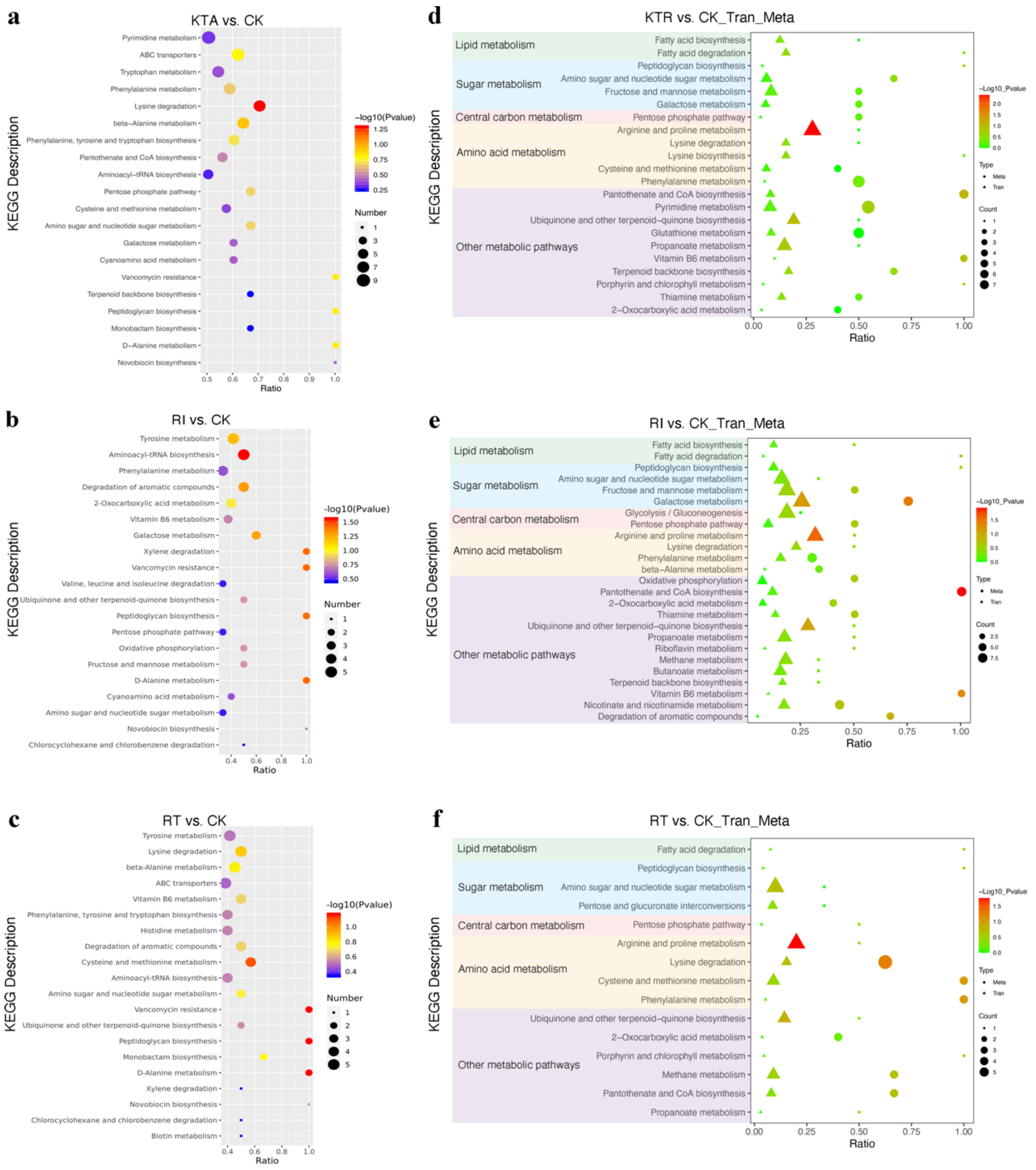

Figure S12. Metabolic perturbation of *E. coli* O157:H7 treated with KTR, RI, and RT. Related to Figure 6.

a-c) KEGG Pathway enrichment analysis of differential metabolites between KTR (a), RI (b), and RT (c) -treated *E. coli* with untreated cells. **d-f)** KEGG Pathway enrichment analysis of integrated transcriptomic and metabolomics data between KTR (d), RI (e), or RT (f) -treated *E. coli* with untreated cells. Significantly enriched pathways were selected to plotting ($P < 0.05$).

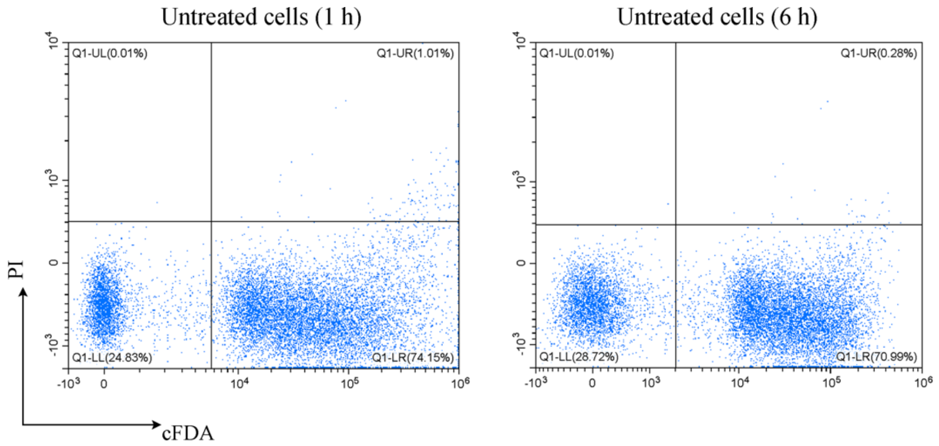

Figure S13. Flow cytometry dot plot of untreated *E. coli* O157:H7 which were stained by cFDA (*x*-axis) and PI (*y*-axis). Related to Figure 5.

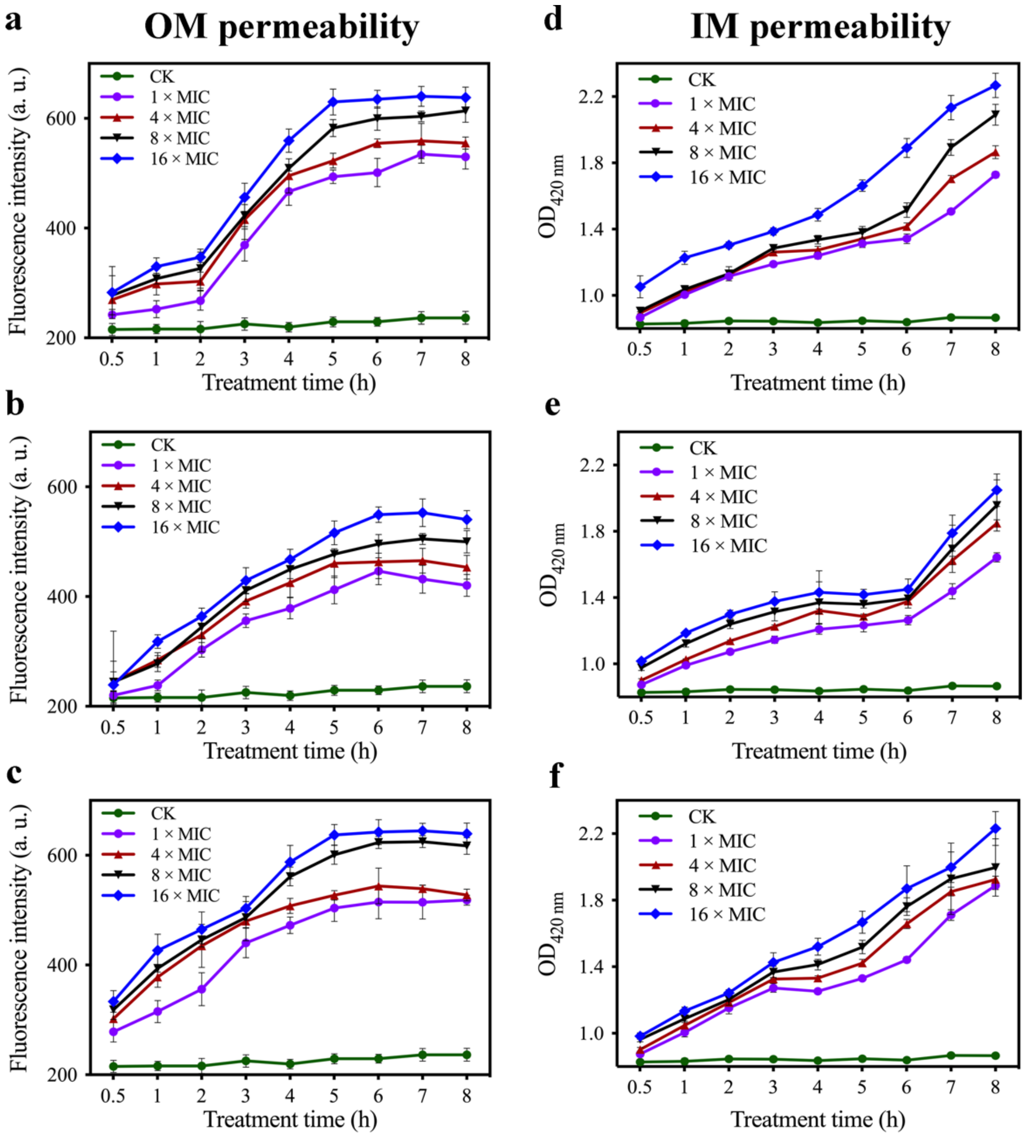

Figure S14. KTR, RI, and RT permeabilize both outer and inner membrane of *E. coli*. Related to Figure 5.

a-c) Dynamic curves of the permeability of the outer membrane probed with NPN. **d-e)** Dynamic curves of the inner membrane permeability of *E. coli* cells, determined optically at 420 nm by measuring the release of cytoplasmic β-galactosidase. *E. coli* cells were treated with 1 ×, 4 ×, 8 ×, and 16 × MIC KTR (a), RI (b), or RT (c, f), respectively. Error bars represent the standard deviation from the mean (n = 3).

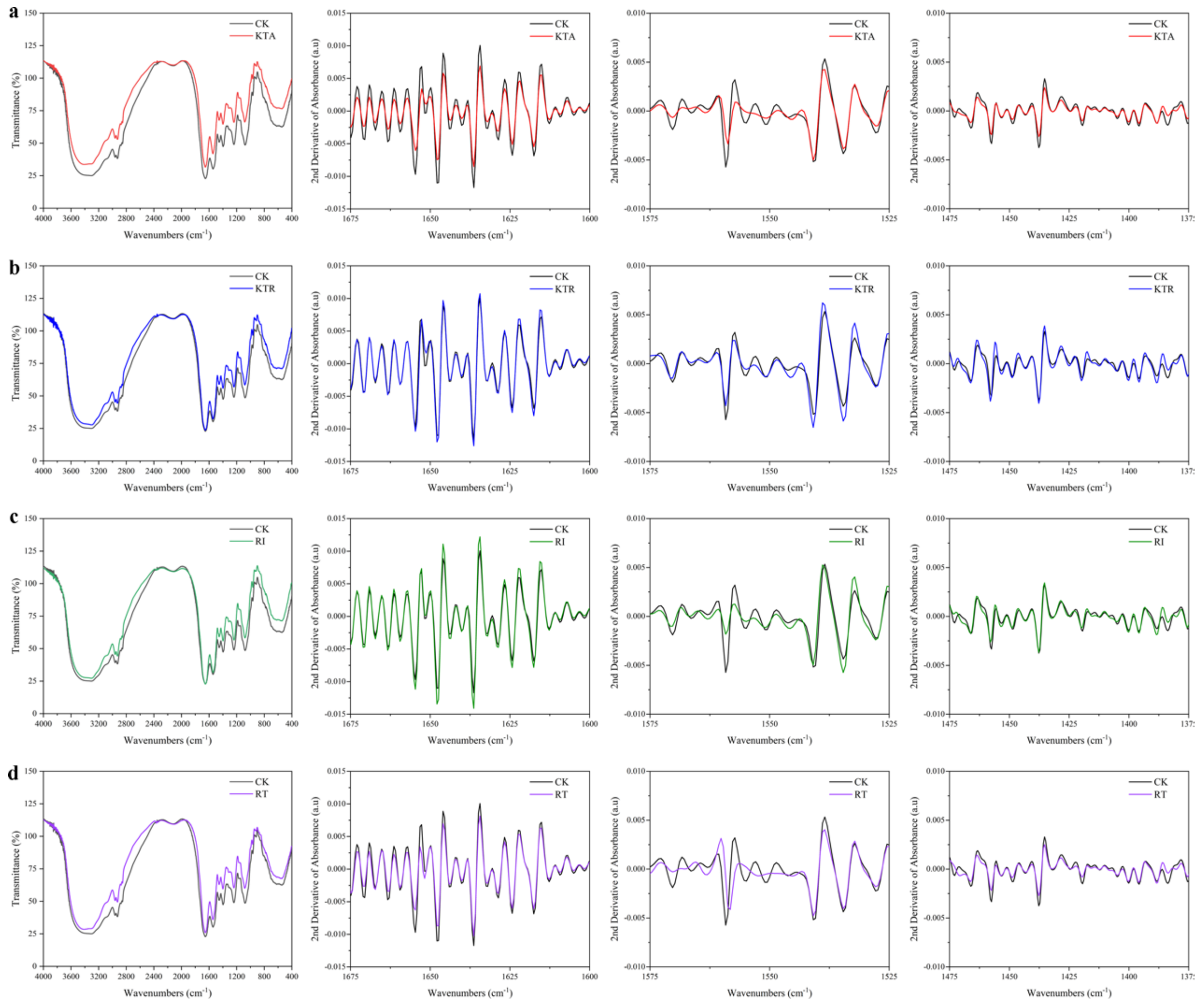

Figure S15. FTIR spectra (4000-400 cm^{-1} wavenumber) and the second derivative of the absorbance (1675-1600 cm^{-1}, 1575-1525 cm^{-1}, and 1475-1375 cm^{-1} wavenumber) of cells treated with KTA (a), KTR (b), RI (c), and RT (d). Related to Figure 5.

E. coli O157:H7 cells were treated with the FCLAPs at 4 × MIC for 1 h. All spectra shown are representative of three independent experiments.

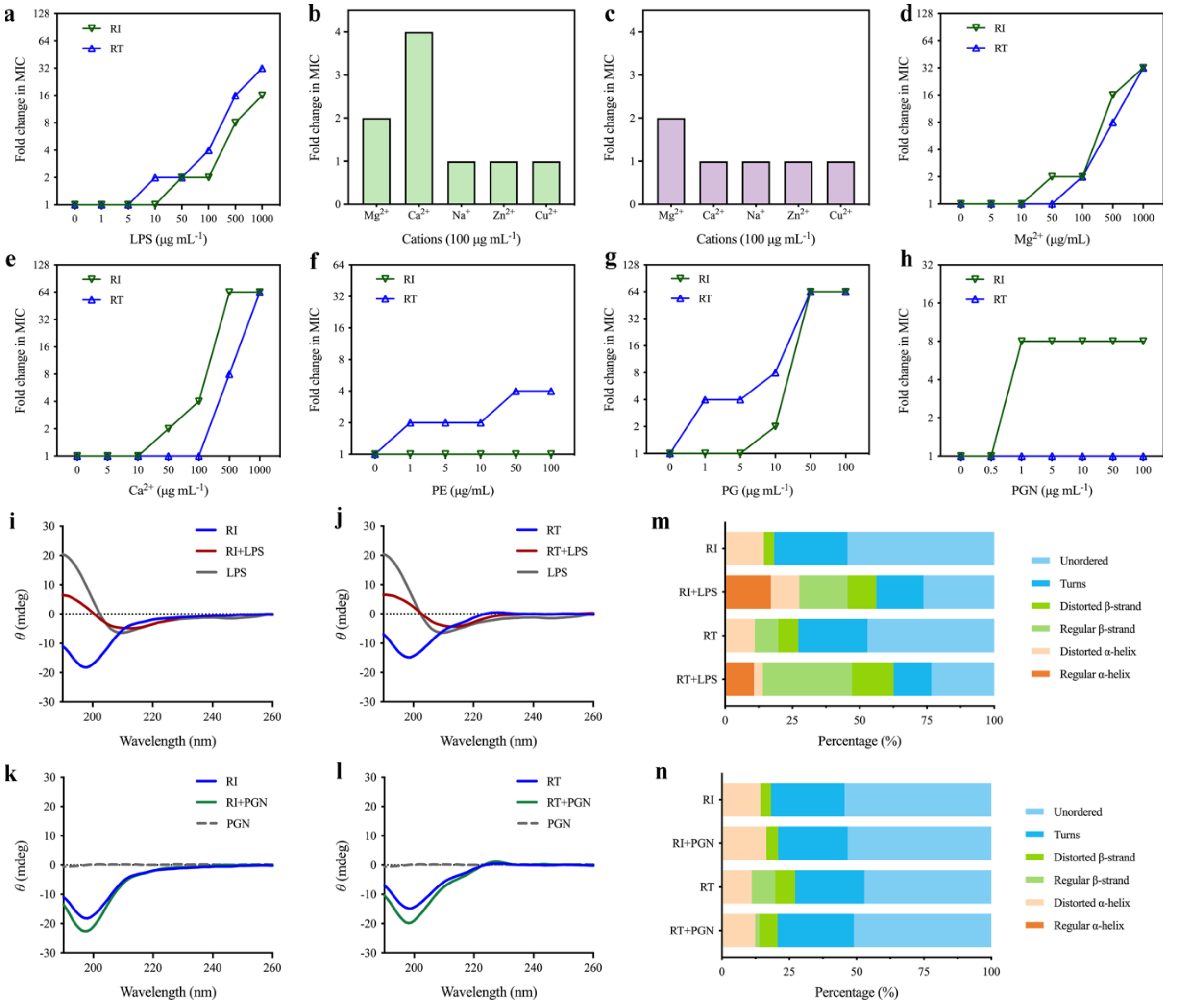

Figure S16. RI and RT exert antibacterial effects through peptide-membrane interaction. Related to Figure 7.

a) Exogenous addition of LPS isolated from *E. coli* O155:B5 impairs the antibacterial activities of RI and RT against *E. coli* O157:H7 in a dose-dependent manner, determined by chequerboard broth microdilution tests. **b-c)** The MIC changes upon exogenous addition of different cations (100 μg mL^{-1} of Mg^{2+}, Ca^{2+}, Na^{+}, Zn^{2+}, Cu^{2+}) to RI and RT, respectively. **d-e)** The MIC changes of RI and RT against *E. coli* O157:H7 in the presence of Mg^{2+} and Ca^{2+}. **f-h)** Exogenous addition of PG, PE, and PGN, respectively, impairs the antibacterial activities of RI and RT against *E. coli* O157:H7 in a dose-dependent manner. **i-j)** Normalized CD spectra of RI and RT (0.1 mmol L^{-1}) in the presence and absence of LPS (25 μmol L^{-1}). **k-l)** Normalized CD spectra of RI and RT (0.1 mmol L^{-1}) in the presence or absence of PGN (1 mmol L^{-1}). **m-n)** Abundance ratio of secondary structure motifs analyzed from CD spectral data.

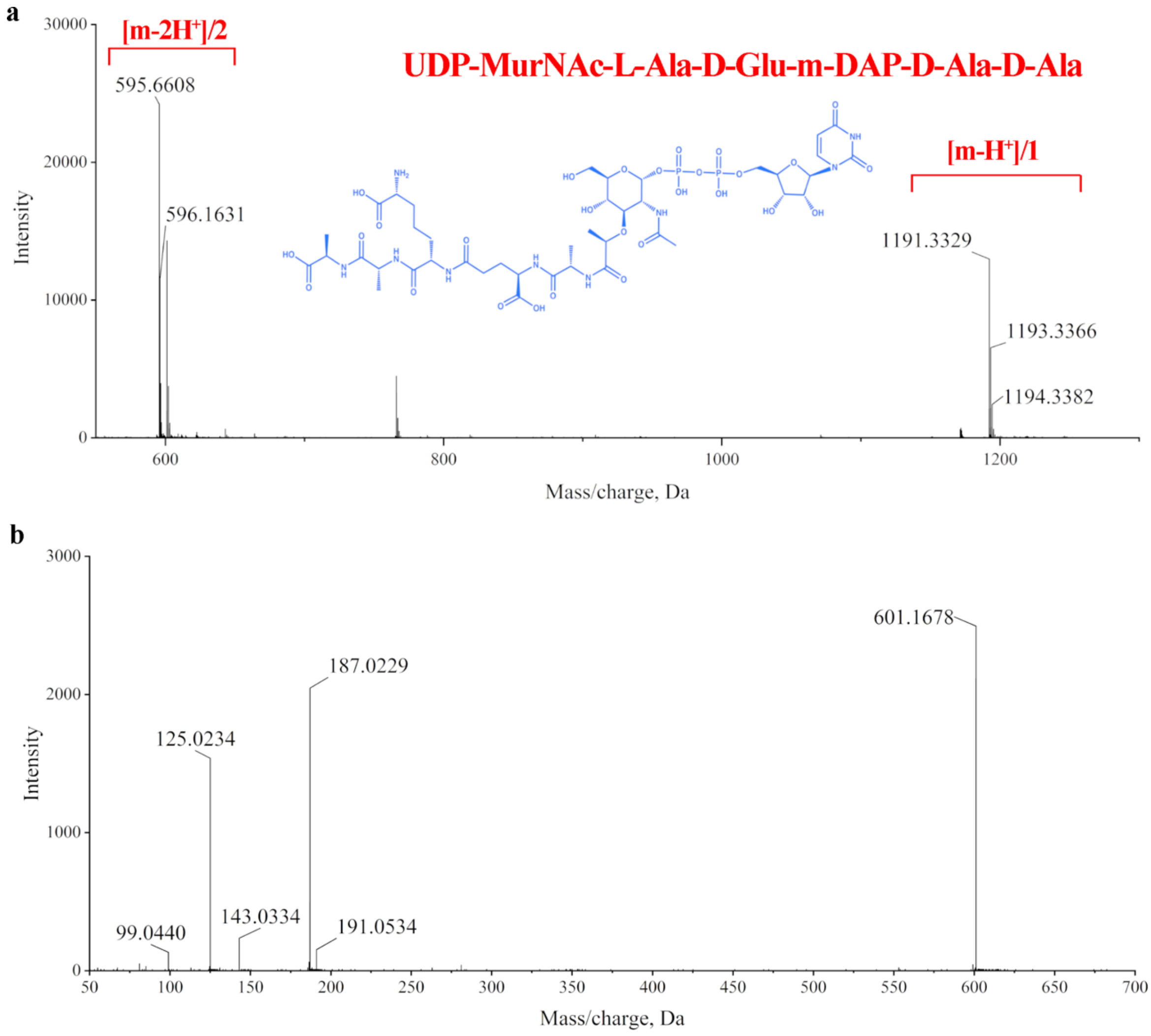

Figure S17. LCMS and LCMS/MS analysis of peptidoglycan precursor UDP-MurNAc-pentapeptide. Related to Figure 8.

a) Mass spectrometry of UDP-MurNAc-L-Ala-D-Glu-m-DAP-D-Ala-D-Ala (UDP-MurNAc-pentapeptide) eluting at 7.33 min. Charge states of singly and doubly ionized molecules are denoted as [m-H$^+$]/1 and [m-2H$^+$]/2. The inset is the structure of UDP-MurNAc-pentapeptide. **b)** The mass spectrum of the fragmentation of the 1192.33 *m/z* ion in the 7.33 min peak. The experiment is representative of 4 independent experiments.
